# Activity of Azole and Non-Azole Substances Against *Aspergillus fumigatus* in Clinical and Environmental Samples to Address Antimicrobial Resistance

**DOI:** 10.3390/ijms26031033

**Published:** 2025-01-25

**Authors:** Isabella Sanseverino, Diletta Scaccabarozzi, Marcos Cuesta Sanz, Miguel Teixeira, Raquel Sabino, Anna Prigitano, Elena Porcel-Rodríguez, Dimitar Marinov, Livia Gómez, Armin Lahm, Luisa Romanò, Teresa Lettieri

**Affiliations:** 1 European Commission, Joint Research Centre (JRC), Via E. Fermi 2749, 21027 Ispra, Italy; isabella.sanseverino@ext.ec.europa.eu (I.S.); diletta.scaccabarozzi@ec.europa.eu (D.S.); marc_cs97@hotmail.com (M.C.S.); miguel.monteiro-teixeira@ec.europa.eu (M.T.); elena.porcel-rodriguez@ec.europa.eu (E.P.-R.); livia.gomez-cortes@ext.ec.europa.eu (L.G.); 2 Faculdade de Farmácia, University of Lisbon, 1649-003 Lisbon, Portugal; raquel.sabino@ff.ulisboa.pt; 3 Instituto de Saúde Ambiental, Faculdade de Medicina, University of Lisbon, 1649-028 Lisbon, Portugal; 4 Laboratório Associado TERRA, Laboratório para o Uso Sustentável da Terra e dos Serviços dos Ecossistemas, Instituto Superior de Agronomia, 1349-017 Lisbon, Portugal; 5 Department of Biomedical Sciences for Health, Università degli Studi di Milano, 20133 Milano, Italy; anna.prigitano@unimi.it (A.P.); luisa.romano@unimi.it (L.R.); 6 Advanced and Reliable Information Systems (ARHS) Developments S.A., 4370 Belvaux, Luxembourg; dimitar.marinov@ext.ec.europa.eu; 7 Bioinformatics Project Support, Piazza Santa Maria Liberatrice 18, 00153 Roma, Italy; armin.lahm@gmail.com

**Keywords:** *Aspergillus fumigatus*, antifungal resistance, *cyp51A* gene, *cyp51B* gene, TR_34_/L98H mutation, azole compounds, environment

## Abstract

*Aspergillus fumigatus* is a common fungus which has gained attention due to its resistance to azole compounds, substances used in both medical and agricultural settings. One of the genetic alterations responsible for this resistance is the mutation TR_34_/L98H in the *cyp51A* gene. The aim of this study was to understand the impact of azoles and non-azoles on *Aspergillus fumigatus*. By examining clinical samples, soil samples, and compost material, this research aims to provide insights into the susceptibility of these strains to antifungal substances. To deepen our understanding of the factors potentially involved in antifungal resistance, we combined in vitro studies of sixteen compounds against *Aspergillus fumigatus* with results from the sequencing of the *cyp51* gene. We observed that compounds generally displayed a similar pattern activity against wild-type *Aspergillus fumigatus*. Non-azoles, except Pyrisoxazole and Amisulbrom, did not show any activity against *Aspergillus fumigatus*, while azole compounds displayed differential activity against the fungus, except for Tetraconazole. For the mutant strains, a generally similar activity was observed in both clinical and environmental samples, likely due to the same mutation in all the isolates. The implications of these findings may be relevant for better understanding the relationship between *Aspergillus fumigatus* and its ability to develop resistance to antifungal substances.

## 1. Introduction

*Aspergillus fumigatus* (*A. fumigatus*), a ubiquitous filamentous fungus, plays a complex role in both environmental and human health. Commonly found in a wide range of habitats, it has garnered significant attention in recent years due to its emerging resistance to azole compounds, crucial antifungal agents in both clinical and agricultural contexts [1,2]. It can be naturally found in many different substrates such as soils, compost, water supply systems, plants, seeds or air leading to cross-resistance development when azole compounds are abundant [3,4]. It is an opportunistic pathogen that can cause a range of diseases in humans, particularly in those with weakened immune systems but also in subjects undergoing immunosuppressive therapy for various conditions, e.g., organ transplant and cancer patients. Azole-resistant *A. fumigatus* has been reported most frequently in Europe, but it represents an increasing global health problem and is now observed at the worldwide scale [4]. The rise of this resistance has increased concerns globally since agricultural practices and environmental reservoirs may serve as potential breeding grounds for these drug-resistant fungi, ultimately affecting human health [4,5,6]. Considering this issue, in 2022, the European Food Safety Authority (EFSA) received, from the European Commission, an interagency mandate referring to the request for a scientific report on the impact of the use of azole fungicides, other than as human medicines, on the development of azole-resistant *Aspergillus* spp., involving five European agencies (the European Centre for Disease Prevention and Control—ECDC, the European Chemicals Agency—ECHA, the European Environment Agency—EEA, the European Food Safety Authority—EFSA, and the European Medicines Agency—EMA), with the support of the Joint Research Centre (JRC) [7].

Medical azole antifungals and azole fungicides share the same mechanism of action: they impact an important step of the ergosterol biosynthetic pathway [8]. Ergosterol is a major sterol in the fungal cell membrane and plays a vital role in maintaining membrane integrity and function. The TR_34_/L98H and TR_46_/Y121F/T289A mutations in the *cyp51A* gene are the most common mutations conferring this azole resistance and both of them are specific genetic alterations associated with environmental resistance selection [1,4,9,10]. The *cyp51A* gene encodes the enzyme lanosterol 14-alpha demethylase, an essential component of the ergosterol biosynthetic pathway in fungi. Azole antifungal drugs target the lanosterol 14-alpha demethylase enzyme, which results in the inhibition of ergosterol biosynthesis, leading to a weakened and disrupted fungal cell membrane [3]. The mutations TR_34_ and L98H occur together in *A. fumigatus* [11,12,13]. The TR_34_ mutation is the insertion of a 34-base pair sequence into the promoter of the *cyp51A* gene which leads to an overexpression of the *cyp51A* [14]. The L98H mutation involves a substitution of the amino acid leucine (L) with histidine (H) at position 98 in the enzyme. This altered amino acid sequence results in a reduced affinity for azole drugs due to a change in the conformational structure of the enzyme [15]. In this study, we have investigated if *A. fumigatus* strains isolated from clinical samples, soil samples, and compost material are susceptible or not to selected azole and non-azole substances with the aim to add new thoughts on how these compounds could affect the incidence of antifungal resistance in Europe. Given the growing concern surrounding antifungal resistance worldwide, this analysis can provide valuable insights into potential contributing factors to resistance and, ultimately, aid in the development of effective strategies to address this one health problem. In the European context, a group of ten azole fungicides (Clotrimazole, Fluconazole, Imazalil, Ipconazole, Metconazole, Miconazole, Penconazole, Prochloraz, Tebuconazole, and Tetraconazole) was recently included in the 3rd Watch List (WL) [16,17,18] and in the 4th WL [19], due to their hazardous properties and potential contribution to antimicrobial resistance (AMR). The selection of antimicrobial pharmaceuticals (including antifungal agents) is also in line with the European One Health Action Plan against antimicrobial resistance [20]. Furthermore, in 2022, the World Health Organisation (WHO) published a priority fungal pathogens list, which categorises *A. fumigatus* in the critical priority group, with the aim of improving the overall response to priority fungal pathogens, including preventing the development of antifungal drug resistance [2]. Additional azoles have be added in the 5th WL [21] for the monitoring of fungicides in the surface water of European Union countries. The surface water WL is indeed a mechanism that under the European Water Framework Directive (WFD) aims at obtaining high-quality monitoring data on emerging pollutants and substances that may represent a significant risk at the Union level to or via the aquatic environment but for which the monitoring data are not sufficient to draw conclusions on the risk they pose.

Following the One Health European Plan, this study will delve into the intricate relationship between *A. fumigatus* and its ability to develop resistance to azole compounds and other antifungal substances, particularly in the context of European agriculture. This investigation will help better understand the molecular aspects of the mutations, the contributing factors to their emergence, and the broader implications for both human health and environmental sustainability. Understanding these dynamics is not only critical for effective strategies in addressing the growing challenge of azole resistance but also for the sustainable use of these antifungals in agriculture, where cross-resistance between medical and agricultural azoles poses a significant concern.

## 2. Results

### 2.1. Clinical A. fumigatus Isolates: Fungicide Activity

Two clinical strains of *A. fumigatus*: wild-type (WT) ATCC 204305 and mutant TR_34_/L98H were used for testing sixteen substances, including azoles and non-azoles: Amisulbrom, Climbazole, Clotrimazole, Fenbuconazole, Fluconazole, Imazalil, Ipconazole, Metconazole, Miconazole, Penconazole, Pyrifenox, Pyrisoxazole, Tebuconazole, Tetraconazole, Triforine, and Prochloraz (see Section 4).

**Figure 1:**
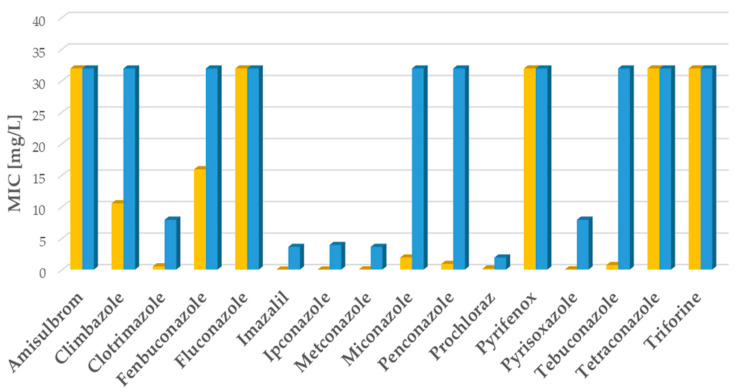
In vitro activity of selected substances against clinical *A. fumigatus* strains. In vitro activity of sixteen substances against the clinical strains *A. fumigatus* ATCC 204305 wild-type (WT) (orange bars) and *A. fumigatus* TR_34_/L98H mutant (blue bars). Data are expressed as minimal inhibitory concentration (MIC) (figure shows the average value over six repetitions for each strain) in mg/L. A total of two strains, one WT and one mutant, were tested.

Eleven substances showed in vitro activity against the clinical strain *A. fumigatus* ATCC 204305 with some differences. Among these substances, nine (Clotrimazole, Imazalil, Ipconazole, Metconazole, Miconazole, Penconazole, Prochloraz, Pyrisoxazole, and Tebuconazole) showed mean minimal inhibitory concentration (MIC) values in the range of 0.10–2 mg/L (Figure 1 and Table S1), and two, Climbazole and Fenbuconazole, displayed weaker activity with mean MIC values of 10.6 mg/L and 16 mg/L, respectively (Figure 1 and Table S1). *A. fumigatus* ATCC 204305 was not susceptible to five substances (Amisulbrom, Fluconazole, Pyrefenox, Tetraconazole, and Triforine) showing MIC values > 32 mg/L (Figure 1 and Table S1). Ten substances (Amisulbrom, Climbazole, Fenbuconazole, Fluconazole, Miconazole, Penconazole, Pyrifenox, Tebuconazole, Tetraconazole, and Triforine) were not active against the clinical mutant *A. fumigatus* TR_34_/L98H (MIC > 32 mg/L), while the remaining substances were active against the mutant with mean MIC values ranging between 2 and 8 mg/L (Figure 1 and Table S1). The six substances Clotrimazole, Imazalil, Ipconazole, Metconazole, Prochloraz, and Pyrisoxazole were effective against both the WT and mutant strains (see Figure 1).

### 2.2. Environmental A. fumigatus Isolates: Fungicide Activity

**Figure 2:**
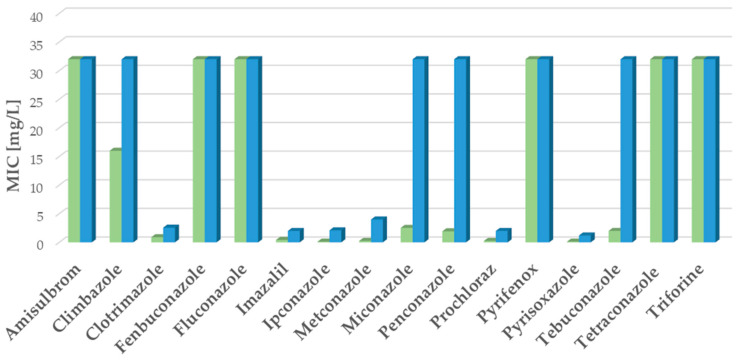
In vitro activity of selected substances against environmental *A. fumigatus* isolates. In vitro activity of sixteen substances against environmental *A. fumigatus* isolates, wild-type (WT, green bars), and mutants (blue bars). Data are expressed as minimal inhibitory concentration (MIC) (average value of six repetitions) in mg/L. Ten isolates were tested: five WT and five mutants.

Sixteen substances (see Section 4) were tested against *A. fumigatus* strains isolated from soil and compost material. The substances Amisulbrom, Fluconazole, Pyrifenox, Tetraconazole, and Triforine were not effective against the WT strains isolated from soil and compost material (Figure 2 and Table S2), as observed in the clinical WT *A. fumigatus* strain ATCC 204305. Fenbuconazole also showed no activity against the WT strains from environmental samples (Figure 2 and Table S2). On the contrary, Clotrimazole, Imazalil, Ipconazole, Metconazole, Penconazole, Prochloraz, Pyrisoxazole, and Tebuconazole were effective against the WT strains, with MIC ranging from 0.062 to 2 mg/L (Figure 2 and Table S2). Miconazole had an average MIC of 2.53 mg/L, while Climbazole exhibited weak activity with a mean MIC of 16 mg/L (Figure 2 and Table S2). Mutant isolates from soil and compost material generally showed similar activity patterns against the tested azoles as the mutant clinical isolates. However, Clotrimazole and Pyrisoxazole had more than two-fold lower MIC values in the environmental isolates compared to the clinical ones (Clotrimazole: mean MIC clinical—8 mg/L and environmental—2.57 mg/L; Pyrisoxazole: mean MIC clinical—8 mg/L and environmental—1.2 mg/L) (Figure 1 and Figure 2, Tables S1 and S2). As observed in clinical strains, Clotrimazole, Imazalil, Ipconazole, Metconazole, Prochloraz, and Pyrisoxazole were effective against both WT and mutant strains in environmental isolates (see Figure 1 and Figure 2).

### 2.3. Relative Susceptibility of Clinical and Environmental A. fumigatus Wild-Type Versus Mutant Strains

**Figure 3:**
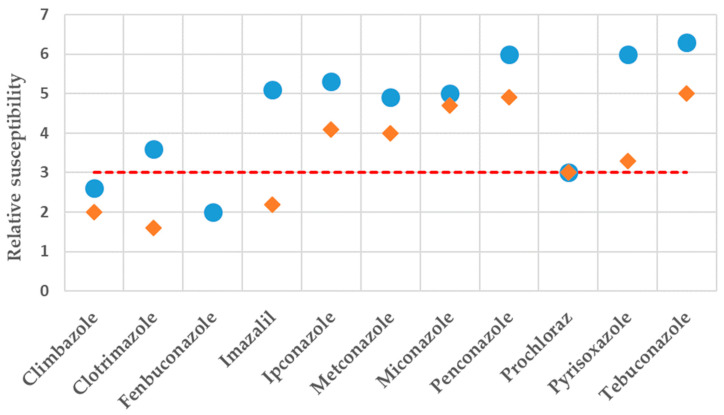
Relative susceptibility for clinical and environmental samples. Relative susceptibility of clinical and environmental mutant strains compared to wild-type (WT) strains determined as log_2_ (mutant MIC)—log_2_ (WT MIC). Only agents with activity against WT isolates are included. The log_2_ difference was not derived for the environmental samples exposed to Fenbuconazole since the WT isolates were not susceptible to this substance. A log_2_ MIC difference ≥ 3 (limit determined by the red dashed line) is considered significant. Blue dots and orange diamonds refer to clinical and environmental samples, respectively.

The relative efficacy of the tested substances against mutant strains versus WT strains is determined as the number of two-fold dilution step differences in concentration that caused complete inhibition of growth of *Aspergillus* (Figure 3 and Table S3). A log_2_ difference of 2.6 and 2 was found for the clinical mutant *A. fumigatus* TR_34_/L98H compared to the clinical WT treated with Climbazole and Fenbuconazole, respectively (Figure 3 and Table S3). The other compounds included in Figure 3 (Clotrimazole, Imazalil, Ipconazole, Metconazole, Miconazole, Penconazole, Prochloraz, Pyrisoxazole, and Tebuconazole) showed a significant log_2_ difference ≥ 3 with Tebuconazole displaying the greatest log_2_ difference (clinical isolates, Figure 3 and Table S3). The environmental isolates showed a significant relative susceptibility (log_2_ difference ≥ 3) of the mutant strains compared to the WT for the substances Ipconazole, Metconazole, Miconazole, Penconazole, Prochloraz, Pyrisoxazole, and Tebuconazole (Figure 3 and Table S3). For Fenbuconazole, the log_2_ difference was not derived for the environmental samples since the WT strains were not susceptible to this substance.

### 2.4. β-Tubulin and Calmodulin Sequencing

For the isolate collected from the compost material (see Section 4), microscopic visualization using Lactophenol Cotton Blue was performed to verify if the isolate belonged to the Fumigati section. In a positive case, species identification was performed using molecular methods based on sequencing partial β-tubulin and calmodulin coding regions. Both sequences were aligned with the WT reference strain *A. fumigatus* ATCC 204305, resulting in a 100% identity score (see Figure S1).

### 2.5. cyp51A and cyp51B Sequencing

Sequencing of the *cyp51A* and *cyp51B* promoter and coding regions was performed to confirm the presence/absence of mutations on the *A. fumigatus* isolates obtained from soil and compost material. All sequences were compared against each other for identification of possible mutations associated with the resistant phenotype (Table 1, and Figures S2 and S3). Three different phenotypes were identified: “wt”, “wt1”, and “mut”. The phenotypes “wt” (Env1, Env2, Env3, Env4) and “wt1” (Env5) contained none of the mutations associated with azole resistance. Although “wt1” did not contain any mutation associated with resistance, it harboured the cyp51A lysine (K) 248 asparagine (N) (K248N) mutation, as well as the cyp51B L42 glutamine (Q) (L42Q) mutation and proline (P) 294P (P294P) mutation. The phenotype “mut” contained the cyp51A azole resistance-associated mutations TR_34_/L98H, K248N, and cyp51B L42Q. Among these mutations, only the TR_34_/L98H has been well described as associated with azole resistance [1,4].

## 3. Discussion

*A. fumigatus* is an airborne pathogenic fungus, commonly found in soil and decaying matter, that can cause serious illnesses, especially in people with a compromised immune system. Diseases caused by *A. fumigatus* are known as aspergillosis, which can range from allergic reactions to severe lung infections, and invasive aspergillosis, when the infection spreads to blood vessels and potentially to other organs. Azoles are widely used in human medicine (e.g., to treat infections caused by *A. fumigatus*), in agriculture (e.g., to protect crops against fungal diseases), as biocides (e.g., for wood preservation), in veterinary medicines (e.g., to treat fungal infections in animals), as industrial chemicals (e.g., as intermediates), and in cosmetics (e.g., as anti-dandruff agents). Their use is associated with the presence of residue concentrations of these substances in different environmental compartments (e.g., surface water, groundwater, sediment) [16,19,22] where they pose a risk to the development and selection of resistant fungal strains, such as *Aspergillus* spp. [22]. In recent years, the increase in number of azole-resistant isolates has gathered alarming attention and it is believed to be linked to the widespread use of azole compounds in both medical treatments and agricultural practices [23]. Scientific evidence shows that azole residues in the environment pose potential toxicity risks to non-target organisms, including changes in community growth [24] and reduced larval growth and development [25]. Azole environmental concentrations exceeding the No Observed Effect Concentration (NOEC) as well as the Predicted No Effect Concentrations (PNEC) are reported in the literature [22]. This evidence suggests that azole resistance development and selection are likely to occur in the environment. However, PNEC values are only based on toxicological data and there is currently no technical guidance for deriving a PNEC that accounts for the contribution to the antifungal resistance in *Aspergillus* spp. So far, the only risk assessment methodology addressing antimicrobial resistance is the PNEC resistance (PNECres) approach developed by Bengtsson-Palme and Larsson [26] and based on MIC data.

Azoles are known to interact with the enzyme cytochrome P450 sterol 14α-demethylase, also known as cyp51, in a non-competitive manner. This interaction prevents the enzyme from removing a methyl group from the precursors of ergosterol, which is a necessary step in the production of ergosterol. As a result, the synthesis of ergosterol, an essential component of fungal cell membranes, is halted [27]. The two variants of the cytochrome P450, cyp51A and cyp51B, may be regulated differentially according to metabolic and environmental needs. Their expression can vary in response to conditions such as the presence of azoles or changes in environmental composition, meaning that one can compensate for the other’s absence [28]. Some studies indicate that azoles may act as competitive inhibitors of the cyp51 enzyme. This means that azoles can interact with the iron atom within the heme group of the enzyme and nearby amino acids in a reversible and competitive manner, thereby challenging the usual substrates for the binding site [29]. Also, in this case, this interaction leads to the accumulation of 14-methylated sterols that affect membrane fluidity and integrity.

In our study, we compared the susceptibility of WT and resistant strains of *A. fumigatus* from both clinical and environmental sources to selected antifungal compounds using MIC experiments following the Clinical Laboratory Standards Institute (CLSI) reference method. MIC is the lowest concentration that prevents visible growth of a strain in vitro. Using the MIC test, we investigated whether *A. fumigatus* strains isolated from clinical, soil, and compost material samples were susceptible or not to selected azole and non-azole substances. The main aim of this study was to investigate how these compounds affect the incidence of antifungal resistance, a growing issue in European countries mainly due to the use of agricultural fungicides in agroecosystems (e.g., to protect crops and fruits against pathogens) and long-term therapies with azole medicines to treat fungal infections, including *Aspergillus*-related diseases. Among the tested substances, nine out of sixteen (Ipconazole, Metconazole, Penconazole, Climbazole, Fenbuconazole, Amisulbrom, Pyrifenox, Pyrisoxazole, and Triforine) have never been tested before for their antifungal effects against *A. fumigatus* in MIC tests. Our results showed that most of the compounds displayed similar activity against *A. fumigatus* WT strains isolated from clinical, soil, and compost material samples. Mutant strains, as expected, reacted differently to the tested azole and non-azole substances in MIC tests compared to the WT strains, except for the compounds showing no activity against *A. fumigatus* WT (Amisulbrom, Fluconazole, Pyrifenox, Tetraconazole, and Triforine for clinical isolates and Amisulbrom, Fenbuconazole, Fluconazole, Pyrifenox, Tetraconazole, and Triforine for environmental isolates). The imidazole Clotrimazole and the pyridine Pyrisoxazole were the only two substances with more than two-fold lower MIC values in mutant strains isolated from soil and compost material, compared to clinical isolates. Fluconazole is not active against filamentous fungi, as confirmed by our data. However, it is used as a treatment for various *Candida* infections. In recent years, a rise in *Candida* spp. resistant to azole compounds has been observed, involving different molecular mechanisms [30]. The constant use of fungicides will continue promoting the emergence of new resistant strains both in the environment and in humans, making treatments ineffective. Additionally, climate change can create an ideal environment for fungal growth and proliferation, particularly due to the higher temperatures and humidity.

To the best of our knowledge, this is the first time Tetraconazole has been tested for its activity against *A. fumigatus* clinical strains using a MIC standardized method. Although it belongs to the triazole class, we observed that Tetraconazole is not active against *A. fumigatus* in either clinical or environmental strains. This agrees with Kano et al.’s (2014) [31] experiments which revealed that Tetraconazole did not induce resistance in *A. fumigatus* isolated from soil when it is used at standard dosages and in low frequencies.

No information is available in the literature on MIC tests performed on *A. fumigatus* using Pyrisoxazole. In our study, this compound, belonging to the class of pyridines, was active against *A. fumigatus*, as demonstrated by our MIC results using clinical, soil, and compost material samples. Despite not being a classical azole, Pyrisoxazole may possess a mechanism of action that affects the sterol biosynthesis in *A. fumigatus* [32]. Further studies, including Surface Plasmon Resonance (SPR) [33,34], to investigate molecular interactions of compounds with specific receptors, are therefore needed to verify possible bindings of Pyrisoxazole to the lanosterol 14alpha-demethylase (cyp51). These studies will also help in understanding why Pyrisoxazole and five other substances (Clotrimazole, Imazalil, Ipconazole, Metconazole, and Prochloraz), showing the same mode of action [35,36], were effective against both WT and mutant strains (TR_34_/L98H) isolated from clinical and environmental sources, despite a reduced activity in TR_34_/L98H being observed. Results comparable to those observed in our studies were also described by Jørgensen et al. [37] for Imazalil, Metconazole, and Prochloraz, suggesting that conformational changes in the cyp51 enzyme caused by the TR_34_/L98H mutations do not significantly impact the binding activity of these substances, allowing them to remain active against the fungus. Furthermore, computational analysis could provide more hints into the structure of the tested compounds when complexed with both WT and mutant *A. fumigatus* cyp51 proteins to investigate the role of *cyp51* mutations in azole-resistance as previously determined for other azole substances [38].

Resistance mutations characterized by tandem repeats (TRs) in the promoter of the *cyp51* gene in combination with single or point mutations (e.g., TR_34_/L98H and TR_46_/Y121F/T289A) are the most common detected in azole-resistant environmental strains of *A. fumigatus.* The evidence that clinical drug-resistant genotypes identified in azole-naïve patients and in patients under chronic azole therapy closely match environmental strains, suggests an environmental route of resistance transmission through exposure of azoles in agricultural practices.

The promoter TR_34_ mutation leads to a higher expression of the *cyp51A*, while the L98H amino acid substitutions result in a reduced affinity for azoles due to a change in the conformational structure of the enzyme [14,15].

In the present study, all isolates harbouring these mutations present an azole resistant phenotype. This finding may partially explain the generally similar activity of the selected compounds against the mutant strains of *A. fumigatus* isolated from clinical, soil, and compost material samples. Looking at the relative susceptibility to the selected compounds, a distinct pattern was particularly observed for Clotrimazole (log_2_ difference of 3.6 for clinical isolates and 1.6 for environmental isolates) and Imazalil (log_2_ difference of 5.1 for clinical isolates and 2.4 for environmental isolates), suggesting these two substances are potential drivers of resistance in clinical but not environmental strains of *A. fumigatus*. For Fenbuconazole, the log_2_ difference was derived only for the clinical samples (log_2_ difference of 2) since WT environmental *A. fumigatus* isolates were not susceptible to this substance, at least at the concentrations checked in this study. All the remaining substances showed a significant log_2_ difference, except Climbazole.

Regarding mutations in *cyp51A*, the one leading to the amino acid substitution N248K was previously identified in an *A. fumigatus* isolated from a patient receiving long-term Voriconazole treatment. However, via the replacement of asparagine 248 by lysine (K248N), no effect occurred on their susceptibility to azoles [39]. Here, the same is observed, with isolates containing either the asparagine 248 (Env1, Env2, Env3, and Env4) or the lysine 248 (Env5) showing similar azole susceptibility profiles, suggesting that Lys248 is not associated with azole resistance.

Although the involvement of cyp51B in azole resistance has not been fully elucidated, an increasing body of evidence points to an important role for this enzyme in azole resistance. The cyp51B G457S mutation was recently associated with *A. fumigatus* resistance to Voriconazole, and suggestions have been made of a possible role in maintaining some level of resistance when cyp51A function is compromised [40,41]. The urgency in understanding cyp51B’s involvement in azole resistance requires more consistent monitoring of this enzyme. For this reason, we decided to include *cyp51B* analysis in the present study. We found the cyp51B L42Q mutation on more than half of the isolates and P394P only in the Env5 isolate. Both mutations were already reported on Itraconazole resistant *A. fumigatus* clinical isolates; however, none were associated with resistance [42]. Here, we confirm that these two mutations are not involved in resistance to azoles, as the Env5 isolate contains both mutations and exhibits an azole-susceptible phenotype.

In conclusion, our study, based on MIC tests, shows that non-azole substances, except for Pyrisoxazole and Amisulbrom, do not exhibit any activity against *A. fumigatus*, suggesting they are unlikely to drive resistance. In contrast, azole compounds display differential activity against the fungus, with the exception of Tetraconazole. MIC tests are therefore envisaged for screening the ability of azole and non-azole substances to induce or select for resistance in *A. fumigatus* and the use of a mixture of fungicides (azoles and non-azoles) in these test should be envisaged to better simulate a field scenario. Furthermore, more studies are needed to understand the mode of action of selected fungicides and to detect new mutations responsible for resistance. Given the increasing global health concern observed at the worldwide scale, this analysis can provide valuable insights into potential contributing factors to resistance and, ultimately, aid in the development of effective strategies to address this issue.

## 4. Materials and Methods

### 4.1. Aspergillus fumigatus Clinical and Environmental Strains

The clinical strains *A. fumigatus* WT ATCC 204305 and *A. fumigatus* TR_34_/L98H mutant were provided by Raquel Sabino from Instituto de Saúde Ambiental, Faculdade de Medicina da Universidade de Lisboa (Lisboa, Portugal). The clinical isolate TR_34_/L98H was collected from a bronchial lavage.

The environmental strains (Env1, Env2, Env3, Env4, Env6, Env7, Env8, Env9, and Env10) were provided by the Medical Mycology Laboratory of Università degli Studi di Milano (Italy) (Table 2). Strains were stored at −80 °C until further use. A volume of 100 µL of the strains was plated on Sabouraud Dextrose Agar (SDA) plates (Sigma-Aldrich, St. Louis, MO, USA). Incubation conditions were 27 °C during 5–7 days or until good sporulation was observed. A negative control was included to ensure the sterility of the SDA plates.

### 4.2. Aspergillus fumigatus Environmental Isolate

A compost material (Env5) was provided by Bas Zwaan from Wageningen University & Research (Wageningen, The Netherlands) and was stored at 2–8 °C until further use (Table 2).

The *A. fumigatus* strain was isolated from the environmental soil sample using Sabouraud Glucose Agar with Chloramphenicol (Sigma-Aldrich, St. Louis, MO, USA) according to the method previously described by Snelders et al. [12], with some modifications. Briefly, a quantity of 2 g of environmental soil was suspended in 15 mL of sterile distilled water, with one drop of Tween 20 and 1 mL of 8 g/L solution of Chloramphenicol (Sigma-Aldrich, St. Louis, MO, USA). After vortexing for 1 min and storing the suspension at room temperature for 30–60 min, 200 µL of the supernatant was plated on the Sabouraud Glucose Agar with Chloramphenicol plates and incubated at 35 °C for 1–3 days. Microscopic visualization was conducted to verify the presence of *A. fumigatus* on the plates using Lactophenol Cotton Blue (Sigma-Aldrich, St. Louis, MO, USA) as dye. 

**Table 2:** *A. fumigatus* strains isolated from soil samples from different regions of Italy and subjected to different agricultural practices. Different types of crops were selected: viticulture, field crops, fruit production, vegetable crops, and flowers. The compost material named Env5 was used for isolating *A. fumigatus.* Env stands for environmental. WT stands for wild-type.

Environmental Sample Name	Environmental Strain Type	Sample Source	Region (District)	Field Treatment
Env1	WT	Corn field	Friuli-Tauriano di Spilimbergo (Pordenone, Italy)	Not known
Env2	WT	Atropa belladonna field (in the botanical garden in the centre of the city)	Marche (Urbino city, Italy)	None
Env3	WT	Vineyard (Valpolicella winery)	Venetian, Santi’Ambrogio della Valpolicella (Verona, Italy)	Other fungicides, not triazoles
Env4	WT	Daffodil bulb pot compost (vase from the home of a cystic fibrosis patient with azole-resistant *A. fumigatus* infection)	Lombardy (Milano area, Italy)	Not known
Env5	WT	Compost material (decaying leaves and grass)	The Netherlands	No azole treatment
Env6	Mutant	Apple orchard (small apple producer)	Lombardy, Valtellina (Sondrio, Italy)	Triazoles
Env7	Mutant	Asparagus field	Apulia, Serracapriola (Foggia, Italy)	Not known
Env8	Mutant	Orchard	Sicily (Caltanissetta, Italy)	None
Env9	Mutant	Orchard (Private orchard in the courtyard)	Tuscany, Lunigiana (Massa, Italy)	None
Env10	Mutant	Plant material waste	Hillegom, The Netherlands	Azole treatment (no specified)

### 4.3. Substances

The substances tested in this study included azole compounds used as Plant Protection Products (PPPs) (Amisulbrom, Imazalil, Ipconazole, Metconazole, Penconazole, Prochloraz, Tebuconazole, and Tetraconazole); azoles used as pharmaceuticals (Clotrimazole, Fluconazole, and Miconazole); azoles used as industrial chemicals (Climbazole and Fenbuconazole); and non-azole substances (Pyrifenox, Pyrisoxazole, and Triforine) used as PPPs. Fluconazole was used as the negative control since *Aspergillus* is intrinsically resistant to this antifungal. Clotrimozole and Miconazole were obtained from the European Pharmacopoeia (EP) Reference Standard. The rest of the substances were purchased from Pestanal^®^ (Merck KGaA, Darmstadt, Germany) (Amisulbrom, Climbazole, Fenbucoazole, Imazalil, Metconazole, Penconazole, Prochloraz, Pyrifenox, Tebuconazole, Tetraconazole, and Triforine), Sigma-Aldrich (St. Louis, MO, USA) (Voriconazole and Fluconazole), and LGC (Teddington, UK) (Ipconazole and Pyrisoxazole). Antifungal stocks were prepared following the M38-A2 protocol from the CLSI (CLSI M38-A2, 2008). The powder was dissolved in DMSO under aseptic conditions and the stocks were sterilized through filtration and the aliquots were stored at −80 °C. Working solutions were freshly prepared the day of the MIC.

### 4.4. Antifungal Susceptibility Test

The M38-A2 protocol from the CLSI (CLSI M38-A2, 2008) [43] was applied for determining the MIC of the environmental isolates for all the selected substances. *A. fumigatus* clinical and environmental isolates were used in the MIC test. The final concentration of the drugs in the test ranged from 32 to 0.03125 µg/mL. Microplates were incubated at 35 °C and examined after 48 h. Two technical replicates were performed for all the substances. The MIC tests were performed in triplicate. The lowest concentration without visible growth was used as the MIC. The effect of Voriconazole on *A. fumigatus* ATCC 204305 was selected as the positive control [44,45]. *A. flavus* ATCC 204304 was used as the internal quality control (CLSI M38-A2, 2008 [43]).

### 4.5. Sanger Sequencing of β-Tubulin and Calmodulin Coding Regions

*A. fumigatus* genomic DNA from the compost material (Env5), isolated by the JRC, was extracted using the ZymoBIOMICS DNA/RNA Miniprep Kit (Zymo Research, Irvine, CA, USA), the quality was determined by Nanodrop and quantified by Qubit^®^ Broad Range Assay Kit, following the manufacturers’ protocol. DNA was stored at −20 °C until further analysis. β-tubulin and calmodulin coding sequences were amplified by PCR using the primers described in [6,46], respectively.

The PCR was performed in 50 µL reaction volume containing 0.25 µL AmpliTaq Gold DNA Polymerase (ThermoFischer Scientific, Waltham, MA, USA), 5 µL of 10 × PCR Buffer II, 3 µL MgCl_2_ (25 mM), 1.25 µL dNTPs (10 mM), 0.3 µL BSA (50 mg/mL), 2 µL of both forward and reverse primers (10 µM), 35.2 µL of nuclease-free water, and 1 µL of genomic DNA (25 ng/µL). Amplification was carried out using the Bio-Rad C1000 Touch thermal cycler (Bio-Rad, Hercules, CA, USA) as follows: 10 min at 95 °C, followed by 35 cycles of 30 s at 95 °C, 30 s at 55 °C, 2 min at 72 °C for β-tubulin and 1 min for calmodulin, and a final extension of 5 min at 72 °C. PCR products were purified using the QIAquick PCR Purification Kit (QIAGEN, Hilden, Germany) and quality was determined by Nanodrop. The purified products were sequenced by Sanger sequencing (Eurofins Genomics, Ebersberg, Germany) and sequences were compared against a reference strain (*A. fumigatus* ATCC 204305, accession numbers KU897017 (β-tubulin) and LC589318 (calmodulin)).

### 4.6. Oxford Nanopore Technologies Sequencing of cyp51A and cyp51B Promoter and Coding Regions

The *cyp51A* and *cyp51B* promoter and coding sequences were amplified by PCR using the following set of primers: *cyp51A*_Fw (5′–TCATATGTTGCTCAGCGG–3′) [47]; *cyp51A*_Rev (5′–TACACCTATTCCGATCACACCA–3′) [48]; *cyp51B*_Fw (5′–ACTAGGGTCAGTTAG–3′) (designed by the JRC); and *cyp51B*_Rev (5′–TCAGGCTTTGGTAGCGG–3′) [49].

The chosen primers amplify a 2136 bp segment corresponding to region 2133603 to 2135738 on contig 10 (*cyp51A*) and a 2061 bp segment corresponding to region 908910 to 910970 on contig 1 (*cyp51B*) of the A. fumigatus ATCC 204305 strain genome assembly e12311eb32db4138, respectively (https://genomes.atcc.org/genomes/d1ada62ecd36451f?tab=overview-tab, accessed on 9 January 2025). Each PCR was performed in replicate in 50 µL reaction volume containing 0.25 µL AmpliTaq Gold DNA Polymerase (ThermoFischer Scientific, Waltham, MA, USA), 5 µL of 10 × PCR Buffer II, 3 µL MgCl_2_ (25 mM), 1.25 µL dNTPs (10 mM), 0.3 µL BSA (50 mg/mL), 3 µL of both forward and reverse primers (10 µM), 32.9 µL of nuclease-free water, and 1 µL of genomic DNA (25 ng/µL). Amplification was carried out using the Bio-Rad C1000 Touch thermal cycler (Bio-Rad, Hercules, CA, USA) as follows: 5 min at 95 °C, followed by 35 cycles of 45 s at 94 °C, 45 s at 58 °C, 2 min at 72 °C, and a final extension of 7 min at 72 °C. PCR products were analysed by gel electrophoresis in 1% agarose gel using the Mid-range DNA Ladder (Invitrogen, Carlsbad, CA, USA) to determine the approximate size of the amplicons. The replicates were pooled together before being purified with the QIAquick PCR Purification Kit (QIAGEN, Hilden, Germany) and the purified DNA quality was determined by Nanodrop. The samples were then sequenced by Oxford Nanopore Technology (ONT, Eurofins Genomics, Ebersberg, Germany) and sequences were processed with the Amplicon Analysis Pipeline v2.4.0 to generate, for each sample, a consensus sequence. The consensus sequences for each gene were aligned using ClustalX v2.0 and manually examined using GeneDoc v2.7. Introns in *cyp51A* and *cyp51B* were removed according to the *A. fumigatus* genome gene annotation (NCBI assembly GCF_000002655.1) and the protein coding part of the resulting alignment was translated. Mutations in the nucleotide or protein alignment were highlighted with GeneDoc v2.7 using the “Conservation Mode” shading option (black background indicating a completely conserved position, grey or white background indicating positions with more than one nucleotide or amino acid present).

### 4.7. Data Analysis

In MIC tests, the average value and standard deviation over six repetitions were calculated for WT and mutant strains. Relative susceptibility of clinical and environmental mutant strains compared to WT strains was determined as log_2_ (mutant MIC)-log_2_ (WT MIC) [37]. A log_2_ MIC difference ≥ 3 was considered significant.

## Figures and Tables

**Table 1:** The detected mutations in the *cyp51* gene and the related phenotype. The results from the alignment of the *cyp51A* and *cyp51B* sequences obtained from the *A. fumigatus* isolates from soil and compost material. Env stands for environmental isolate. TR strands for tandem repeats. L stands for leucine, H for histidine, K for lysine, N for asparagine, Q for glutamine, and P for proline. * Silent mutation (G1182T).

		Env1	Env2	Env3	Env4	Env5	Env6	Env7	Env8	Env9	Env10
*cyp51A*	TR_34_						+	+	+	+	+
	L98H						+	+	+	+	+
	K248N					+	+	+	+	+	+
*cyp51B*	L42Q					+	+	+	+	+	+
	P394P *					+					
Phenotype		wt	wt	wt	wt	wt1	mut	mut	mut	mut	mut

## Data Availability

The original contributions presented in this study are included in the article/Supplementary Materials. Further inquiries can be directed to the corresponding author.

## Supplementary Materials

The following supporting information can be downloaded at: https://www.mdpi.com/article/10.3390/ijms26031033/s1.
