# Supplementary material for: Activity of Azole and Non-Azole Substances Against Aspergillus fumigatus in Clinical and Environmental Samples to Address Antimicrobial Resistance
Source: Int J Mol Sci. 2025 Jan 25;26(3):1033. doi: 10.3390/ijms26031033 (PMC11816432; doi:10.3390/ijms26031033)
Supplement: Supplementary file 1 [file ijms-26-01033-s001.zip › ijms-3407053-supplementary.pdf]

Supplementary Materials

Table S1. ***In vitro* activity of selected substances against clinical *Aspergillus fumigatus* strains**. *In vitro* activity of sixteen substances against the clinical strains *Aspergillus fumigatus (A. fumigatus)* ATCC 204305 wild-type (WT) and *A. fumigatus* TR_{34}/L98H mutant. Data are expressed as minimal inhibitory concentration (MIC) (table shows the average value over six repetitions for each strain and in brackets, the minimum and maximum value) in mg/L. A total of two strains, one WT and one mutant, were tested. SD stands for standard deviation.

Substances	Clinical *A. fumigatus* ATCC 204305 WT	Clinical *A. fumigatus* TR_{34}/L98H	SD *A. fumigatus* ATCC 204305 WT	SD *A. fumigatus* TR_{34}/L98H
**Amisulbrom**	>32	>32	0	0
**Climbazole**	10.6 (8-16)	>32	4.1	0
**Clotrimazole**	0.6 (0.5-1)	8	0.26	0
**Fenbuconazole**	16	>32	0	0
**Fluconazole**	>32	>32	0	0
**Imazalil**	0.10 (0.062-0.125)	3.7 (2-4)	0.03	0.82
**Ipconazole**	0.10 (0.062-0.125)	4	0.03	0
**Metconazole**	0.12 (0.062-0.25)	3.7 (2-4)	0.1	0.82
**Miconazole**	2	>32	0	0
**Penconazole**	1	>32	0	0
**Prochloraz**	0.25	2	0	0
**Pyrifenox**	>32	>32	0	0
**Pyrisoxazole**	0.12	8	0	0
**Tebuconazole**	0.8 (0.5-1)	>32	0.26	0
**Tetraconazole**	>32	>32	0	0
**Triforine**	>32	>32	0	0

Table S2. ***In vitro* activity of selected substances against environmental *Aspergillus fumigatus* isolates**. *In vitro* activity of 16 substances against the environmental isolates *Aspergillus fumigatus (A. fumigatus),* wild-type (WT) and mutants. Data are expressed as minimal inhibitory concentration (MIC) (average value and range of six repetitions) in mg/L. SD stands for standard deviation. n stands for number of isolates.

Substances	Environmental *A. fumigatus* WT n = 5	Environmental *A. fumigatus* TR_{34}/L98H n = 5	SD *A. fumigatus* WT	SD *A. fumigatus* TR_{34}/L98H
**Amisulbrom**	>32	>32	0	0
**Climbazole**	16	>32	0	0
**Clotrimazole**	0.9 (0.5-1)	2.57 (2-8)	0.2	1.62
**Fenbuconazole**	>32	>32	0	0
**Fluconazole**	>32	>32	0	0
**Imazalil**	0.43 (0.1-0.5)	2	0.15	0
**Ipconazole**	0.12 (0.062-0.125)	2.1 (1-4)	0.02	0.82
**Metconazole**	0.25	4	0	0
**Miconazole**	2.53 (2-4)	>32	0.9	0
**Penconazole**	1.93 (1-2)	>32	0.25	14

**Prochloraz**	0.25	2	0	0
**Pyrifenox**	>32	>32	0	0
**Pyrisoxazole**	0.13	1.2 (1-2)	0	0.41
**Tebuconazole**	2	>32	0	0
**Tetraconazole**	>32	>32	0	0
**Triforine**	>32	>32	0	0

Table S3. **Relative susceptibility for clinical and environmental samples.** Relative susceptibility of clinical and environmental mutant strains compared to wild-type (WT) strains determined as $\log_2$ (mutant MIC) – $\log_2$ (WT MIC). Only agents with activity against WT isolates are included. The $\log_2$ difference was not derived for environmental samples exposed to Fenbuconazole since the WT strains were not susceptible to this substance. A $\log_2$ MIC difference ≥ 3 is considered significant.

**Substances**	**Clinical *Aspergillus fumigatus* TR$_{34}$/L98H**	**Environmental *Aspergillus fumigatus* mutant**
**Climbazole**	2.6	2
**Clotrimazole**	3.6	1.6
**Fenbuconazole**	2	-
**Imazalil**	5.1	2.2
**Ipconazole**	5.3	4.1
**Metconazole**	4.9	4
**Miconazole**	5	4.7
**Penconazole**	6	4.9
**Prochloraz**	3	3
**Pyrisoxazole**	6	3.3
**Tebuconazole**	6.3	5

Figure S1. Sequence alignment of β-tubulin and Calmodulin. Alignment of the β-tubulin (A) and Calmodulin (B) DNA sequences between the *Aspergillus fumigatus* reference strain ATCC_204305 and JRC's isolate Env5.

A

```
B-tub_ATCC_204305      GGGTGATTGGGATCTCTCATCTTAGCAGGCTACCTCCATGGGTTCAGCCTCACTGTCATG 60
B-tub_Env5             GGGTGATTGGGATCTCTCATCTTAGCAGGCTACCTCCATGGGTTCAGCCTCACTGTCATG 60
                       ************************************************************

B-tub_ATCC_204305      GGTATCAGCTAACAAATCTACAGGCAGACCATCTCTGGTGAGCATGGCCTTGACGGCTCT 120
B-tub_Env5             GGTATCAGCTAACAAATCTACAGGCAGACCATCTCTGGTGAGCATGGCCTTGACGGCTCT 120
                       ************************************************************

B-tub_ATCC_204305      GGCCAGTAAGTTCGACCTATATCCTCCCAATTGAGAAAGCGGCGGAAACACGGAAAACAA 180
B-tub_Env5             GGCCAGTAAGTTCGACCTATATCCTCCCAATTGAGAAAGCGGCGGAAACACGGAAAACAA 180
                       ************************************************************

B-tub_ATCC_204305      GGAAGAAGCGGACGCGTGTCTGATGGGAAATAATAGCTACAATGGCTCCTCCGATCTCCA 240
B-tub_Env5             GGAAGAAGCGGACGCGTGTCTGATGGGAAATAATAGCTACAATGGCTCCTCCGATCTCCA 240
                       ************************************************************

B-tub_ATCC_204305      GCTGGAGCGTATGAACGTCTATTTCAACGAGGTGTGTGGATGAAACTCTTGATTTATACT 300
B-tub_Env5             GCTGGAGCGTATGAACGTCTATTTCAACGAGGTGTGTGGATGAAACTCTTGATTTATACT 300
                       ************************************************************

B-tub_ATCC_204305      ATTTCGGCAACATCTCACGATCTGACTCGCTACTAGGCCAACGGTGACAAATATGTTCCT 360
B-tub_Env5             ATTTCGGCAACATCTCACGATCTGACTCGCTACTAGGCCAACGGTGACAAATATGTTCCT 360
                       ************************************************************

B-tub_ATCC_204305      CGTGCCGTTCTGGTCGATCTCGAGCCTGGTACCATGGACGCTGTCCGTGCCGGTCCCTTC 420
B-tub_Env5             CGTGCCGTTCTGGTCGATCTCGAGCCTGGTACCATGGACGCTGTCCGTGCCGGTCCCTTC 420
                       ************************************************************

B-tub_ATCC_204305      GGCGAGCTATTCCGTCCCGACAACTTCGTCTTCGGCCAGTCCGGTGCTGGTAACAACTGG 480
B-tub_Env5             GGCGAGCTATTCCGTCCCGACAACTTCGTCTTCGGCCAGTCCGGTGCTGGTAACAACTGG 480
                       ************************************************************
```

B

```
Calmo_ATCC_204305      GGTCGTTGTATAGGAGGGATCTCCAGAATATTGAGGGTGTGCGCTGACACGAGATTTGAC 60
Calm_Env5              GGTCGTTGTATAGGAGGGATCTCCAGAATATTGAGGGTGTGCGCTGACACGAGATTTGAC 60
                       ************************************************************

Calmo_ATCC_204305      GTATAGGACAAGGATGGTGATGGTTAGTGACCCTTTTTCCACTCCTCGAACTTCGGCTTC 120
Calm_Env5              GTATAGGACAAGGATGGTGATGGTTAGTGACCCTTTTTCCACTCCTCGAACTTCGGCTTC 120
                       ************************************************************

Calmo_ATCC_204305      CATGCGATCATGTTCAAACGCCGACTCACAATATCCGGAAATGACCCGTCAGTACTGATA 180
Calm_Env5              CATGCGATCATGTTCAAACGCCGACTCACAATATCCGGAAATGACCCGTCAGTACTGATA 180
                       ************************************************************

Calmo_ATCC_204305      ATATCTATGTTTGACTATCAGGCCAGATCACCACCAAGGAATTGGGCACTGTAATGCGCT 240
Calm_Env5              ATATCTATGTTTGACTATCAGGCCAGATCACCACCAAGGAATTGGGCACTGTAATGCGCT 240
                       ************************************************************

Calmo_ATCC_204305      CTCTGGGCCAGAACCCTTCCGAGTCAGAGCTGCAAGATATGATCAACGAGGTGGATGCTG 300
Calm_Env5              CTCTGGGCCAGAACCCTTCCGAGTCAGAGCTGCAAGATATGATCAACGAGGTGGATGCTG 300
                       ************************************************************

Calmo_ATCC_204305      ACAACAACGGCACCATCGATTTCCCCGGTATGTGATACTTTCGGTATGAACTCGGGAGGG 360
Calm_Env5              ACAACAACGGCACCATCGATTTCCCCGGTATGTGATACTTTCGGTATGAACTCGGGAGGG 360
                       ************************************************************

Calmo_ATCC_204305      GAGAGAACAATCATTAACTTGTAATCAGAATTCCTTACCATGATGGCTCGGAAGATGAAG 420
Calm_Env5              GAGAGAACAATCATTAACTTGTAATCAGAATTCCTTACCATGATGGCTCGGAAGATGAAG 420
                       ************************************************************

Calmo_ATCC_204305      GACACCGACTCCGAAGAGGAAATTCGGGAAGCTTTCAAGGTCTTCGACCGCGACAACAAC 480
Calm_Env5              GACACCGACTCCGAAGAGGAAATTCGGGAAGCTTTCAAGGTCTTCGACCGCGACAACAAC 480
                       ************************************************************

Calmo_ATCC_204305      GGTTTCATCTCCGCTGCGGAGCTGCGCCACG 511
Calm_Env5              GGTTTCATCTCCGCTGCGGAGCTGCGCCACG 511
                       *******************************
```

Figure S2. Alignment of the isolated *cyp51A* nucleotide sequences. Location of primers cyp51A_Fw and cyp51A_Rev is indicated. Introns are represented by black bars above the alignment and were assigned according to the known splicing pattern for *cyp51A*. Black background colour indicates positions where identical nucleotides are present.

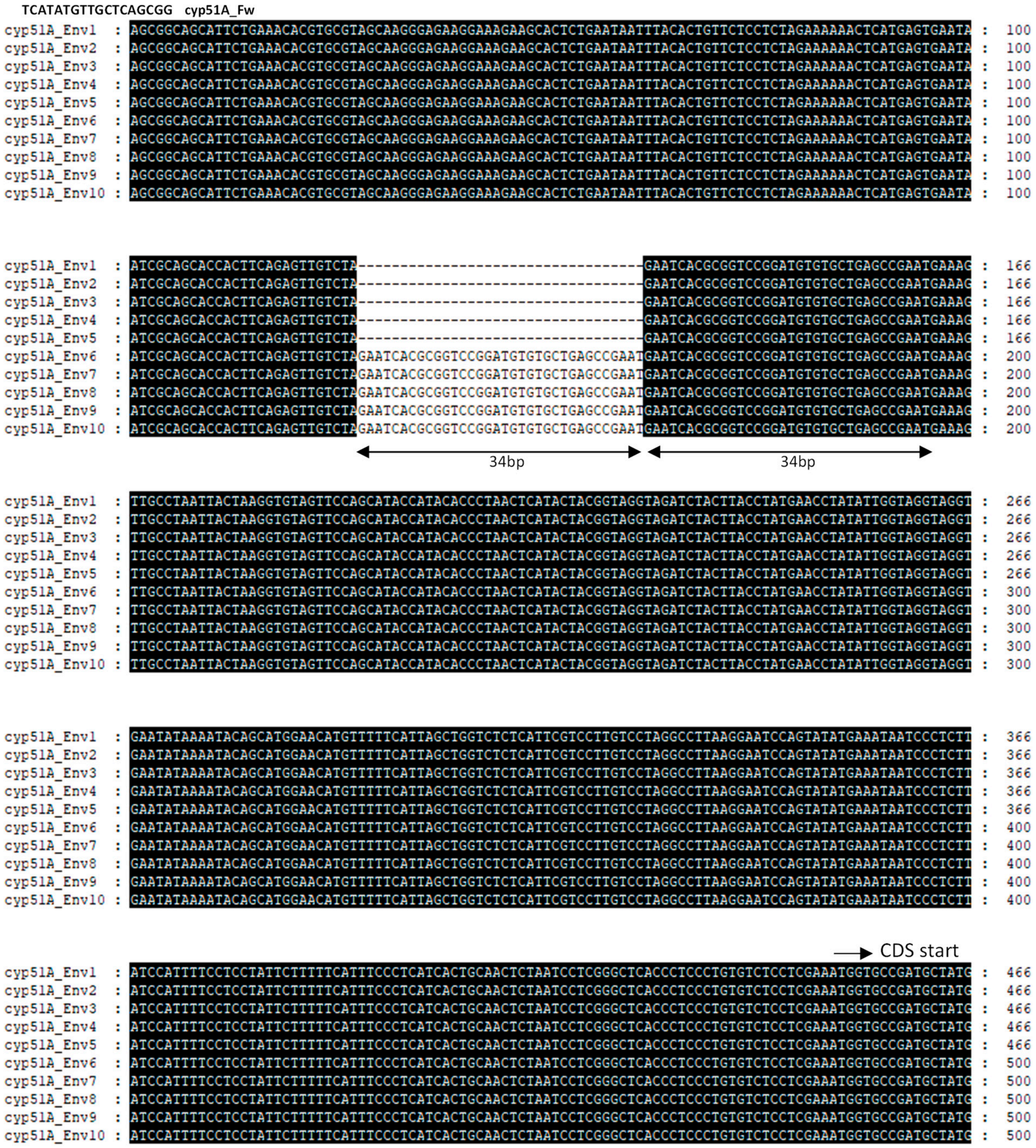

```
cyp51A_Env1  : GCTTACGGCCTACATGGCCGTTGCGGTGCTGACGGCAATCTTGCTCAATGTTGTTTATCAATTATTCTTTCGGCTTTGGAACCGAACAGAACCGCCAATG :  566
cyp51A_Env2  : GCTTACGGCCTACATGGCCGTTGCGGTGCTGACGGCAATCTTGCTCAATGTTGTTTATCAATTATTCTTTCGGCTTTGGAACCGAACAGAACCGCCAATG :  566
cyp51A_Env3  : GCTTACGGCCTACATGGCCGTTGCGGTGCTGACGGCAATCTTGCTCAATGTTGTTTATCAATTATTCTTTCGGCTTTGGAACCGAACAGAACCGCCAATG :  566
cyp51A_Env4  : GCTTACGGCCTACATGGCCGTTGCGGTGCTGACGGCAATCTTGCTCAATGTTGTTTATCAATTATTCTTTCGGCTTTGGAACCGAACAGAACCGCCAATG :  566
cyp51A_Env5  : GCTTACGGCCTACATGGCCGTTGCGGTGCTGACGGCAATCTTGCTCAATGTTGTTTATCAATTATTCTTTCGGCTTTGGAACCGAACAGAACCGCCAATG :  566
cyp51A_Env6  : GCTTACGGCCTACATGGCCGTTGCGGTGCTGACGGCAATCTTGCTCAATGTTGTTTATCAATTATTCTTTCGGCTTTGGAACCGAACAGAACCGCCAATG :  600
cyp51A_Env7  : GCTTACGGCCTACATGGCCGTTGCGGTGCTGACGGCAATCTTGCTCAATGTTGTTTATCAATTATTCTTTCGGCTTTGGAACCGAACAGAACCGCCAATG :  600
cyp51A_Env8  : GCTTACGGCCTACATGGCCGTTGCGGTGCTGACGGCAATCTTGCTCAATGTTGTTTATCAATTATTCTTTCGGCTTTGGAACCGAACAGAACCGCCAATG :  600
cyp51A_Env9  : GCTTACGGCCTACATGGCCGTTGCGGTGCTGACGGCAATCTTGCTCAATGTTGTTTATCAATTATTCTTTCGGCTTTGGAACCGAACAGAACCGCCAATG :  600
cyp51A_Env10 : GCTTACGGCCTACATGGCCGTTGCGGTGCTGACGGCAATCTTGCTCAATGTTGTTTATCAATTATTCTTTCGGCTTTGGAACCGAACAGAACCGCCAATG :  600

cyp51A_Env1  : GTCTTTCATTGGGTCCCATTTCTGGGTAGTACCATCAGTTACGGGATTGATCCCTACAAGTTCTTCTTTGCGTGCAGAGAAAAGGCAAGTCTCAAGATTG :  666
cyp51A_Env2  : GTCTTTCATTGGGTCCCATTTCTGGGTAGTACCATCAGTTACGGGATTGATCCCTACAAGTTCTTCTTTGCGTGCAGAGAAAAGGCAAGTCTCAAGATTG :  666
cyp51A_Env3  : GTCTTTCATTGGGTCCCATTTCTGGGTAGTACCATCAGTTACGGGATTGATCCCTACAAGTTCTTCTTTGCGTGCAGAGAAAAGGCAAGTCTCAAGATTG :  666
cyp51A_Env4  : GTCTTTCATTGGGTCCCATTTCTGGGTAGTACCATCAGTTACGGGATTGATCCCTACAAGTTCTTCTTTGCGTGCAGAGAAAAGGCAAGTCTCAAGATTG :  666
cyp51A_Env5  : GTCTTTCATTGGGTCCCATTTCTGGGTAGTACCATCAGTTACGGGATTGATCCCTACAAGTTCTTCTTTGCGTGCAGAGAAAAGGCAAGTCTCAAGATTG :  666
cyp51A_Env6  : GTCTTTCATTGGGTCCCATTTCTGGGTAGTACCATCAGTTACGGGATTGATCCCTACAAGTTCTTCTTTGCGTGCAGAGAAAAGGCAAGTCTCAAGATTG :  700
cyp51A_Env7  : GTCTTTCATTGGGTCCCATTTCTGGGTAGTACCATCAGTTACGGGATTGATCCCTACAAGTTCTTCTTTGCGTGCAGAGAAAAGGCAAGTCTCAAGATTG :  700
cyp51A_Env8  : GTCTTTCATTGGGTCCCATTTCTGGGTAGTACCATCAGTTACGGGATTGATCCCTACAAGTTCTTCTTTGCGTGCAGAGAAAAGGCAAGTCTCAAGATTG :  700
cyp51A_Env9  : GTCTTTCATTGGGTCCCATTTCTGGGTAGTACCATCAGTTACGGGATTGATCCCTACAAGTTCTTCTTTGCGTGCAGAGAAAAGGCAAGTCTCAAGATTG :  700
cyp51A_Env10 : GTCTTTCATTGGGTCCCATTTCTGGGTAGTACCATCAGTTACGGGATTGATCCCTACAAGTTCTTCTTTGCGTGCAGAGAAAAGGCAAGTCTCAAGATTG :  700

cyp51A_Env1  : TAGTTTGACATTCATTCCTGGGCGCATTGCTGAGTATTGCTTTCTTAACCGGCAGTATGGCGATATCTTCACTTTTATACTGTTGGGTCAAAAAACCACA :  766
cyp51A_Env2  : TAGTTTGACATTCATTCCTGGGCGCATTGCTGAGTATTGCTTTCTTAACCGGCAGTATGGCGATATCTTCACTTTTATACTGTTGGGTCAAAAAACCACA :  766
cyp51A_Env3  : TAGTTTGACATTCATTCCTGGGCGCATTGCTGAGTATTGCTTTCTTAACCGGCAGTATGGCGATATCTTCACTTTTATACTGTTGGGTCAAAAAACCACA :  766
cyp51A_Env4  : TAGTTTGACATTCATTCCTGGGCGCATTGCTGAGTATTGCTTTCTTAACCGGCAGTATGGCGATATCTTCACTTTTATACTGTTGGGTCAAAAAACCACA :  766
cyp51A_Env5  : TAGTTTGACATTCATTCCTGGGCGCATTGCTGAGTATTGCTTTCTTAACCGGCAGTATGGCGATATCTTCACTTTTATACTGTTGGGTCAAAAAACCACA :  766
cyp51A_Env6  : TAGTTTGACATTCATTCCTGGGCGCATTGCTGAGTATTGCTTTCTTAACCGGCAGTATGGCGATATCTTCACTTTTATACTGTTGGGTCAAAAAACCACA :  800
cyp51A_Env7  : TAGTTTGACATTCATTCCTGGGCGCATTGCTGAGTATTGCTTTCTTAACCGGCAGTATGGCGATATCTTCACTTTTATACTGTTGGGTCAAAAAACCACA :  800
cyp51A_Env8  : TAGTTTGACATTCATTCCTGGGCGCATTGCTGAGTATTGCTTTCTTAACCGGCAGTATGGCGATATCTTCACTTTTATACTGTTGGGTCAAAAAACCACA :  800
cyp51A_Env9  : TAGTTTGACATTCATTCCTGGGCGCATTGCTGAGTATTGCTTTCTTAACCGGCAGTATGGCGATATCTTCACTTTTATACTGTTGGGTCAAAAAACCACA :  800
cyp51A_Env10 : TAGTTTGACATTCATTCCTGGGCGCATTGCTGAGTATTGCTTTCTTAACCGGCAGTATGGCGATATCTTCACTTTTATACTGTTGGGTCAAAAAACCACA :  800

                                                         L98H
                                                      CTC->CAC
cyp51A_Env1  : GTCTACCTGGGCGTTCAGGGGAACGAGTTTATTCTCAACGGCAAGCTCAAGGATGTCAATGCGGAAGAGGTCTATAGTCCATTGACGACCCCCGTTTTCG :  866
cyp51A_Env2  : GTCTACCTGGGCGTTCAGGGGAACGAGTTTATTCTCAACGGCAAGCTCAAGGATGTCAATGCGGAAGAGGTCTATAGTCCATTGACGACCCCCGTTTTCG :  866
cyp51A_Env3  : GTCTACCTGGGCGTTCAGGGGAACGAGTTTATTCTCAACGGCAAGCTCAAGGATGTCAATGCGGAAGAGGTCTATAGTCCATTGACGACCCCCGTTTTCG :  866
cyp51A_Env4  : GTCTACCTGGGCGTTCAGGGGAACGAGTTTATTCTCAACGGCAAGCTCAAGGATGTCAATGCGGAAGAGGTCTATAGTCCATTGACGACCCCCGTTTTCG :  866
cyp51A_Env5  : GTCTACCTGGGCGTTCAGGGGAACGAGTTTATTCTCAACGGCAAGCTCAAGGATGTCAATGCGGAAGAGGTCTATAGTCCATTGACGACCCCCGTTTTCG :  866
cyp51A_Env6  : GTCTACCTGGGCGTTCAGGGGAACGAGTTTATTCTCAACGGCAAGCACAAGGATGTCAATGCGGAAGAGGTCTATAGTCCATTGACGACCCCCGTTTTCG :  900
cyp51A_Env7  : GTCTACCTGGGCGTTCAGGGGAACGAGTTTATTCTCAACGGCAAGCACAAGGATGTCAATGCGGAAGAGGTCTATAGTCCATTGACGACCCCCGTTTTCG :  900
cyp51A_Env8  : GTCTACCTGGGCGTTCAGGGGAACGAGTTTATTCTCAACGGCAAGCACAAGGATGTCAATGCGGAAGAGGTCTATAGTCCATTGACGACCCCCGTTTTCG :  900
cyp51A_Env9  : GTCTACCTGGGCGTTCAGGGGAACGAGTTTATTCTCAACGGCAAGCACAAGGATGTCAATGCGGAAGAGGTCTATAGTCCATTGACGACCCCCGTTTTCG :  900
cyp51A_Env10 : GTCTACCTGGGCGTTCAGGGGAACGAGTTTATTCTCAACGGCAAGCACAAGGATGTCAATGCGGAAGAGGTCTATAGTCCATTGACGACCCCCGTTTTCG :  900

cyp51A_Env1  : GATCGGACGTGGTGTATGATTGTCCCAATTCCAAGCTGATGGAGCAGAAAAAGTTCATCAAGTACGGCTTGACTCAGTCTGCGTTAGAGTCTCATGTGCC :  966
cyp51A_Env2  : GATCGGACGTGGTGTATGATTGTCCCAATTCCAAGCTGATGGAGCAGAAAAAGTTCATCAAGTACGGCTTGACTCAGTCTGCGTTAGAGTCTCATGTGCC :  966
cyp51A_Env3  : GATCGGACGTGGTGTATGATTGTCCCAATTCCAAGCTGATGGAGCAGAAAAAGTTCATCAAGTACGGCTTGACTCAGTCTGCGTTAGAGTCTCATGTGCC :  966
cyp51A_Env4  : GATCGGACGTGGTGTATGATTGTCCCAATTCCAAGCTGATGGAGCAGAAAAAGTTCATCAAGTACGGCTTGACTCAGTCTGCGTTAGAGTCTCATGTGCC :  966
cyp51A_Env5  : GATCGGACGTGGTGTATGATTGTCCCAATTCCAAGCTGATGGAGCAGAAAAAGTTCATCAAGTACGGCTTGACTCAGTCTGCGTTAGAGTCTCATGTGCC :  966
cyp51A_Env6  : GATCGGACGTGGTGTATGATTGTCCCAATTCCAAGCTGATGGAGCAGAAAAAGTTCATCAAGTACGGCTTGACTCAGTCTGCGTTAGAGTCTCATGTGCC : 1000
cyp51A_Env7  : GATCGGACGTGGTGTATGATTGTCCCAATTCCAAGCTGATGGAGCAGAAAAAGTTCATCAAGTACGGCTTGACTCAGTCTGCGTTAGAGTCTCATGTGCC : 1000
cyp51A_Env8  : GATCGGACGTGGTGTATGATTGTCCCAATTCCAAGCTGATGGAGCAGAAAAAGTTCATCAAGTACGGCTTGACTCAGTCTGCGTTAGAGTCTCATGTGCC : 1000
cyp51A_Env9  : GATCGGACGTGGTGTATGATTGTCCCAATTCCAAGCTGATGGAGCAGAAAAAGTTCATCAAGTACGGCTTGACTCAGTCTGCGTTAGAGTCTCATGTGCC : 1000
cyp51A_Env10 : GATCGGACGTGGTGTATGATTGTCCCAATTCCAAGCTGATGGAGCAGAAAAAGTTCATCAAGTACGGCTTGACTCAGTCTGCGTTAGAGTCTCATGTGCC : 1000
```

```
cyp51A_Env1  : ACTTATTGAGAAGGAGGTTTTGGACTATCTGCGCGATTCACCGAACTTTCAAGGCTCGTCCGGCCGGATGGACATCTCTGCGGCAATGGCTGAGATTACC : 1066
cyp51A_Env2  : ACTTATTGAGAAGGAGGTTTTGGACTATCTGCGCGATTCACCGAACTTTCAAGGCTCGTCCGGCCGGATGGACATCTCTGCGGCAATGGCTGAGATTACC : 1066
cyp51A_Env3  : ACTTATTGAGAAGGAGGTTTTGGACTATCTGCGCGATTCACCGAACTTTCAAGGCTCGTCCGGCCGGATGGACATCTCTGCGGCAATGGCTGAGATTACC : 1066
cyp51A_Env4  : ACTTATTGAGAAGGAGGTTTTGGACTATCTGCGCGATTCACCGAACTTTCAAGGCTCGTCCGGCCGGATGGACATCTCTGCGGCAATGGCTGAGATTACC : 1066
cyp51A_Env5  : ACTTATTGAGAAGGAGGTTTTGGACTATCTGCGCGATTCACCGAACTTTCAAGGCTCGTCCGGCCGGATGGACATCTCTGCGGCAATGGCTGAGATTACC : 1066
cyp51A_Env6  : ACTTATTGAGAAGGAGGTTTTGGACTATCTGCGCGATTCACCGAACTTTCAAGGCTCGTCCGGCCGGATGGACATCTCTGCGGCAATGGCTGAGATTACC : 1100
cyp51A_Env7  : ACTTATTGAGAAGGAGGTTTTGGACTATCTGCGCGATTCACCGAACTTTCAAGGCTCGTCCGGCCGGATGGACATCTCTGCGGCAATGGCTGAGATTACC : 1100
cyp51A_Env8  : ACTTATTGAGAAGGAGGTTTTGGACTATCTGCGCGATTCACCGAACTTTCAAGGCTCGTCCGGCCGGATGGACATCTCTGCGGCAATGGCTGAGATTACC : 1100
cyp51A_Env9  : ACTTATTGAGAAGGAGGTTTTGGACTATCTGCGCGATTCACCGAACTTTCAAGGCTCGTCCGGCCGGATGGACATCTCTGCGGCAATGGCTGAGATTACC : 1100
cyp51A_Env10 : ACTTATTGAGAAGGAGGTTTTGGACTATCTGCGCGATTCACCGAACTTTCAAGGCTCGTCCGGCCGGATGGACATCTCTGCGGCAATGGCTGAGATTACC : 1100

cyp51A_Env1  : ATTTTTACCGCTGCTCGAGCCCTCCAAGGCCAGGAAGTTCGTTCCAAACTCACGGCTGAGTTCGCTGACCTCTATCATGACCTGGACAAGGGCTTTACTC : 1166
cyp51A_Env2  : ATTTTTACCGCTGCTCGAGCCCTCCAAGGCCAGGAAGTTCGTTCCAAACTCACGGCTGAGTTCGCTGACCTCTATCATGACCTGGACAAGGGCTTTACTC : 1166
cyp51A_Env3  : ATTTTTACCGCTGCTCGAGCCCTCCAAGGCCAGGAAGTTCGTTCCAAACTCACGGCTGAGTTCGCTGACCTCTATCATGACCTGGACAAGGGCTTTACTC : 1166
cyp51A_Env4  : ATTTTTACCGCTGCTCGAGCCCTCCAAGGCCAGGAAGTTCGTTCCAAACTCACGGCTGAGTTCGCTGACCTCTATCATGACCTGGACAAGGGCTTTACTC : 1166
cyp51A_Env5  : ATTTTTACCGCTGCTCGAGCCCTCCAAGGCCAGGAAGTTCGTTCCAAACTCACGGCTGAGTTCGCTGACCTCTATCATGACCTGGACAAGGGCTTTACTC : 1166
cyp51A_Env6  : ATTTTTACCGCTGCTCGAGCCCTCCAAGGCCAGGAAGTTCGTTCCAAACTCACGGCTGAGTTCGCTGACCTCTATCATGACCTGGACAAGGGCTTTACTC : 1200
cyp51A_Env7  : ATTTTTACCGCTGCTCGAGCCCTCCAAGGCCAGGAAGTTCGTTCCAAACTCACGGCTGAGTTCGCTGACCTCTATCATGACCTGGACAAGGGCTTTACTC : 1200
cyp51A_Env8  : ATTTTTACCGCTGCTCGAGCCCTCCAAGGCCAGGAAGTTCGTTCCAAACTCACGGCTGAGTTCGCTGACCTCTATCATGACCTGGACAAGGGCTTTACTC : 1200
cyp51A_Env9  : ATTTTTACCGCTGCTCGAGCCCTCCAAGGCCAGGAAGTTCGTTCCAAACTCACGGCTGAGTTCGCTGACCTCTATCATGACCTGGACAAGGGCTTTACTC : 1200
cyp51A_Env10 : ATTTTTACCGCTGCTCGAGCCCTCCAAGGCCAGGAAGTTCGTTCCAAACTCACGGCTGAGTTCGCTGACCTCTATCATGACCTGGACAAGGGCTTTACTC : 1200

                                                                                                               K248N
                                                                                                             AAA->AAT
cyp51A_Env1  : CCATCAATTTTATGCTACCGTGGGCCCCATTGCCGCATAACAAGAAGCGAGATGCTGCTCATGCGCGCATGAGGTCAATCTACGTTGACATCATCAAACA : 1266
cyp51A_Env2  : CCATCAATTTTATGCTACCGTGGGCCCCATTGCCGCATAACAAGAAGCGAGATGCTGCTCATGCGCGCATGAGGTCAATCTACGTTGACATCATCAAACA : 1266
cyp51A_Env3  : CCATCAATTTTATGCTACCGTGGGCCCCATTGCCGCATAACAAGAAGCGAGATGCTGCTCATGCGCGCATGAGGTCAATCTACGTTGACATCATCAAACA : 1266
cyp51A_Env4  : CCATCAATTTTATGCTACCGTGGGCCCCATTGCCGCATAACAAGAAGCGAGATGCTGCTCATGCGCGCATGAGGTCAATCTACGTTGACATCATCAAACA : 1266
cyp51A_Env5  : CCATCAATTTTATGCTACCGTGGGCCCCATTGCCGCATAACAAGAAGCGAGATGCTGCTCATGCGCGCATGAGGTCAATCTACGTTGACATCATCAATCA : 1266
cyp51A_Env6  : CCATCAATTTTATGCTACCGTGGGCCCCATTGCCGCATAACAAGAAGCGAGATGCTGCTCATGCGCGCATGAGGTCAATCTACGTTGACATCATCAATCA : 1300
cyp51A_Env7  : CCATCAATTTTATGCTACCGTGGGCCCCATTGCCGCATAACAAGAAGCGAGATGCTGCTCATGCGCGCATGAGGTCAATCTACGTTGACATCATCAATCA : 1300
cyp51A_Env8  : CCATCAATTTTATGCTACCGTGGGCCCCATTGCCGCATAACAAGAAGCGAGATGCTGCTCATGCGCGCATGAGGTCAATCTACGTTGACATCATCAATCA : 1300
cyp51A_Env9  : CCATCAATTTTATGCTACCGTGGGCCCCATTGCCGCATAACAAGAAGCGAGATGCTGCTCATGCGCGCATGAGGTCAATCTACGTTGACATCATCAATCA : 1300
cyp51A_Env10 : CCATCAATTTTATGCTACCGTGGGCCCCATTGCCGCATAACAAGAAGCGAGATGCTGCTCATGCGCGCATGAGGTCAATCTACGTTGACATCATCAATCA : 1300

cyp51A_Env1  : GCGCCGTCTTGACGGTGACAAGGACTCTCAGAAATCAGACATGATATGGAACCTGATGAACTGCACATACAAAAACGGCCAGCAAGTGCCTGATAAAGAG : 1366
cyp51A_Env2  : GCGCCGTCTTGACGGTGACAAGGACTCTCAGAAATCAGACATGATATGGAACCTGATGAACTGCACATACAAAAACGGCCAGCAAGTGCCTGATAAAGAG : 1366
cyp51A_Env3  : GCGCCGTCTTGACGGTGACAAGGACTCTCAGAAATCAGACATGATATGGAACCTGATGAACTGCACATACAAAAACGGCCAGCAAGTGCCTGATAAAGAG : 1366
cyp51A_Env4  : GCGCCGTCTTGACGGTGACAAGGACTCTCAGAAATCAGACATGATATGGAACCTGATGAACTGCACATACAAAAACGGCCAGCAAGTGCCTGATAAAGAG : 1366
cyp51A_Env5  : GCGCCGTCTTGACGGTGACAAGGACTCTCAGAAATCAGACATGATATGGAACCTGATGAACTGCACATACAAAAACGGCCAGCAAGTGCCTGATAAAGAG : 1366
cyp51A_Env6  : GCGCCGTCTTGACGGTGACAAGGACTCTCAGAAATCAGACATGATATGGAACCTGATGAACTGCACATACAAAAACGGCCAGCAAGTGCCTGATAAAGAG : 1400
cyp51A_Env7  : GCGCCGTCTTGACGGTGACAAGGACTCTCAGAAATCAGACATGATATGGAACCTGATGAACTGCACATACAAAAACGGCCAGCAAGTGCCTGATAAAGAG : 1400
cyp51A_Env8  : GCGCCGTCTTGACGGTGACAAGGACTCTCAGAAATCAGACATGATATGGAACCTGATGAACTGCACATACAAAAACGGCCAGCAAGTGCCTGATAAAGAG : 1400
cyp51A_Env9  : GCGCCGTCTTGACGGTGACAAGGACTCTCAGAAATCAGACATGATATGGAACCTGATGAACTGCACATACAAAAACGGCCAGCAAGTGCCTGATAAAGAG : 1400
cyp51A_Env10 : GCGCCGTCTTGACGGTGACAAGGACTCTCAGAAATCAGACATGATATGGAACCTGATGAACTGCACATACAAAAACGGCCAGCAAGTGCCTGATAAAGAG : 1400

cyp51A_Env1  : ATTGCGCACATGATGATAACCCTGTTGATGGCTGGTCAGCATTCGTCTTCGTCCATCAGCGCCTGGATTATGCTGAGACTGGCCTCACAGCCAAAAGTCC : 1466
cyp51A_Env2  : ATTGCGCACATGATGATAACCCTGTTGATGGCTGGTCAGCATTCGTCTTCGTCCATCAGCGCCTGGATTATGCTGAGACTGGCCTCACAGCCAAAAGTCC : 1466
cyp51A_Env3  : ATTGCGCACATGATGATAACCCTGTTGATGGCTGGTCAGCATTCGTCTTCGTCCATCAGCGCCTGGATTATGCTGAGACTGGCCTCACAGCCAAAAGTCC : 1466
cyp51A_Env4  : ATTGCGCACATGATGATAACCCTGTTGATGGCTGGTCAGCATTCGTCTTCGTCCATCAGCGCCTGGATTATGCTGAGACTGGCCTCACAGCCAAAAGTCC : 1466
cyp51A_Env5  : ATTGCGCACATGATGATAACCCTGTTGATGGCTGGTCAGCATTCGTCTTCGTCCATCAGCGCCTGGATTATGCTGAGACTGGCCTCACAGCCAAAAGTCC : 1466
cyp51A_Env6  : ATTGCGCACATGATGATAACCCTGTTGATGGCTGGTCAGCATTCGTCTTCGTCCATCAGCGCCTGGATTATGCTGAGACTGGCCTCACAGCCAAAAGTCC : 1500
cyp51A_Env7  : ATTGCGCACATGATGATAACCCTGTTGATGGCTGGTCAGCATTCGTCTTCGTCCATCAGCGCCTGGATTATGCTGAGACTGGCCTCACAGCCAAAAGTCC : 1500
cyp51A_Env8  : ATTGCGCACATGATGATAACCCTGTTGATGGCTGGTCAGCATTCGTCTTCGTCCATCAGCGCCTGGATTATGCTGAGACTGGCCTCACAGCCAAAAGTCC : 1500
cyp51A_Env9  : ATTGCGCACATGATGATAACCCTGTTGATGGCTGGTCAGCATTCGTCTTCGTCCATCAGCGCCTGGATTATGCTGAGACTGGCCTCACAGCCAAAAGTCC : 1500
cyp51A_Env10 : ATTGCGCACATGATGATAACCCTGTTGATGGCTGGTCAGCATTCGTCTTCGTCCATCAGCGCCTGGATTATGCTGAGACTGGCCTCACAGCCAAAAGTCC : 1500
```

```
cyp51A_Env1  : TCGAAGAGCTGTATCAGGAACAGCTGGCCAATCTTGGCCCCGCCGGGCCAGACGGCAGTCTTCCTCCGCTCCAGTACAAGGATCTTGACAAACTTCCCTT : 1566
cyp51A_Env2  : TCGAAGAGCTGTATCAGGAACAGCTGGCCAATCTTGGCCCCGCCGGGCCAGACGGCAGTCTTCCTCCGCTCCAGTACAAGGATCTTGACAAACTTCCCTT : 1566
cyp51A_Env3  : TCGAAGAGCTGTATCAGGAACAGCTGGCCAATCTTGGCCCCGCCGGGCCAGACGGCAGTCTTCCTCCGCTCCAGTACAAGGATCTTGACAAACTTCCCTT : 1566
cyp51A_Env4  : TCGAAGAGCTGTATCAGGAACAGCTGGCCAATCTTGGCCCCGCCGGGCCAGACGGCAGTCTTCCTCCGCTCCAGTACAAGGATCTTGACAAACTTCCCTT : 1566
cyp51A_Env5  : TCGAAGAGCTGTATCAGGAACAGCTGGCCAATCTTGGCCCCGCCGGGCCAGACGGCAGTCTTCCTCCGCTCCAGTACAAGGATCTTGACAAACTTCCCTT : 1566
cyp51A_Env6  : TCGAAGAGCTGTATCAGGAACAGCTGGCCAATCTTGGCCCCGCCGGGCCAGACGGCAGTCTTCCTCCGCTCCAGTACAAGGATCTTGACAAACTTCCCTT : 1600
cyp51A_Env7  : TCGAAGAGCTGTATCAGGAACAGCTGGCCAATCTTGGCCCCGCCGGGCCAGACGGCAGTCTTCCTCCGCTCCAGTACAAGGATCTTGACAAACTTCCCTT : 1600
cyp51A_Env8  : TCGAAGAGCTGTATCAGGAACAGCTGGCCAATCTTGGCCCCGCCGGGCCAGACGGCAGTCTTCCTCCGCTCCAGTACAAGGATCTTGACAAACTTCCCTT : 1600
cyp51A_Env9  : TCGAAGAGCTGTATCAGGAACAGCTGGCCAATCTTGGCCCCGCCGGGCCAGACGGCAGTCTTCCTCCGCTCCAGTACAAGGATCTTGACAAACTTCCCTT : 1600
cyp51A_Env10 : TCGAAGAGCTGTATCAGGAACAGCTGGCCAATCTTGGCCCCGCCGGGCCAGACGGCAGTCTTCCTCCGCTCCAGTACAAGGATCTTGACAAACTTCCCTT : 1600

cyp51A_Env1  : CCATCAACATGTTATTCGTGAAACCTTACGGATTCACTCCTCTATTCACTCTATCATGCGCAAGGTGAAAAGCCCCTTGCCCGTTCCCGGGACCCCTTAC : 1666
cyp51A_Env2  : CCATCAACATGTTATTCGTGAAACCTTACGGATTCACTCCTCTATTCACTCTATCATGCGCAAGGTGAAAAGCCCCTTGCCCGTTCCCGGGACCCCTTAC : 1666
cyp51A_Env3  : CCATCAACATGTTATTCGTGAAACCTTACGGATTCACTCCTCTATTCACTCTATCATGCGCAAGGTGAAAAGCCCCTTGCCCGTTCCCGGGACCCCTTAC : 1666
cyp51A_Env4  : CCATCAACATGTTATTCGTGAAACCTTACGGATTCACTCCTCTATTCACTCTATCATGCGCAAGGTGAAAAGCCCCTTGCCCGTTCCCGGGACCCCTTAC : 1666
cyp51A_Env5  : CCATCAACATGTTATTCGTGAAACCTTACGGATTCACTCCTCTATTCACTCTATCATGCGCAAGGTGAAAAGCCCCTTGCCCGTTCCCGGGACCCCTTAC : 1666
cyp51A_Env6  : CCATCAACATGTTATTCGTGAAACCTTACGGATTCACTCCTCTATTCACTCTATCATGCGCAAGGTGAAAAGCCCCTTGCCCGTTCCCGGGACCCCTTAC : 1700
cyp51A_Env7  : CCATCAACATGTTATTCGTGAAACCTTACGGATTCACTCCTCTATTCACTCTATCATGCGCAAGGTGAAAAGCCCCTTGCCCGTTCCCGGGACCCCTTAC : 1700
cyp51A_Env8  : CCATCAACATGTTATTCGTGAAACCTTACGGATTCACTCCTCTATTCACTCTATCATGCGCAAGGTGAAAAGCCCCTTGCCCGTTCCCGGGACCCCTTAC : 1700
cyp51A_Env9  : CCATCAACATGTTATTCGTGAAACCTTACGGATTCACTCCTCTATTCACTCTATCATGCGCAAGGTGAAAAGCCCCTTGCCCGTTCCCGGGACCCCTTAC : 1700
cyp51A_Env10 : CCATCAACATGTTATTCGTGAAACCTTACGGATTCACTCCTCTATTCACTCTATCATGCGCAAGGTGAAAAGCCCCTTGCCCGTTCCCGGGACCCCTTAC : 1700

cyp51A_Env1  : ATGATTCCTCCCGGTCGCGTGCTCCTTGCTTCACCTGGAGTGACAGCCCTCAGCGACGAACACTTCCCCAATGCTGGGTGCTGGGATCCCCATCGCTGGG : 1766
cyp51A_Env2  : ATGATTCCTCCCGGTCGCGTGCTCCTTGCTTCACCTGGAGTGACAGCCCTCAGCGACGAACACTTCCCCAATGCTGGGTGCTGGGATCCCCATCGCTGGG : 1766
cyp51A_Env3  : ATGATTCCTCCCGGTCGCGTGCTCCTTGCTTCACCTGGAGTGACAGCCCTCAGCGACGAACACTTCCCCAATGCTGGGTGCTGGGATCCCCATCGCTGGG : 1766
cyp51A_Env4  : ATGATTCCTCCCGGTCGCGTGCTCCTTGCTTCACCTGGAGTGACAGCCCTCAGCGACGAACACTTCCCCAATGCTGGGTGCTGGGATCCCCATCGCTGGG : 1766
cyp51A_Env5  : ATGATTCCTCCCGGTCGCGTGCTCCTTGCTTCACCTGGAGTGACAGCCCTCAGCGACGAACACTTCCCCAATGCTGGGTGCTGGGATCCCCATCGCTGGG : 1766
cyp51A_Env6  : ATGATTCCTCCCGGTCGCGTGCTCCTTGCTTCACCTGGAGTGACAGCCCTCAGCGACGAACACTTCCCCAATGCTGGGTGCTGGGATCCCCATCGCTGGG : 1800
cyp51A_Env7  : ATGATTCCTCCCGGTCGCGTGCTCCTTGCTTCACCTGGAGTGACAGCCCTCAGCGACGAACACTTCCCCAATGCTGGGTGCTGGGATCCCCATCGCTGGG : 1800
cyp51A_Env8  : ATGATTCCTCCCGGTCGCGTGCTCCTTGCTTCACCTGGAGTGACAGCCCTCAGCGACGAACACTTCCCCAATGCTGGGTGCTGGGATCCCCATCGCTGGG : 1800
cyp51A_Env9  : ATGATTCCTCCCGGTCGCGTGCTCCTTGCTTCACCTGGAGTGACAGCCCTCAGCGACGAACACTTCCCCAATGCTGGGTGCTGGGATCCCCATCGCTGGG : 1800
cyp51A_Env10 : ATGATTCCTCCCGGTCGCGTGCTCCTTGCTTCACCTGGAGTGACAGCCCTCAGCGACGAACACTTCCCCAATGCTGGGTGCTGGGATCCCCATCGCTGGG : 1800

cyp51A_Env1  : AGAACCAGGCTACTAAGGAGCAGGAGAACGACGAGGTTGTCGACTACGGTTACGGCGCCGTCTCCAAGGGCACGTCAAGTCCCTATCTTCCGTTTGGTGC : 1866
cyp51A_Env2  : AGAACCAGGCTACTAAGGAGCAGGAGAACGACGAGGTTGTCGACTACGGTTACGGCGCCGTCTCCAAGGGCACGTCAAGTCCCTATCTTCCGTTTGGTGC : 1866
cyp51A_Env3  : AGAACCAGGCTACTAAGGAGCAGGAGAACGACGAGGTTGTCGACTACGGTTACGGCGCCGTCTCCAAGGGCACGTCAAGTCCCTATCTTCCGTTTGGTGC : 1866
cyp51A_Env4  : AGAACCAGGCTACTAAGGAGCAGGAGAACGACGAGGTTGTCGACTACGGTTACGGCGCCGTCTCCAAGGGCACGTCAAGTCCCTATCTTCCGTTTGGTGC : 1866
cyp51A_Env5  : AGAACCAGGCTACTAAGGAGCAGGAGAACGACGAGGTTGTCGACTACGGTTACGGCGCCGTCTCCAAGGGCACGTCAAGTCCCTATCTTCCGTTTGGTGC : 1866
cyp51A_Env6  : AGAACCAGGCTACTAAGGAGCAGGAGAACGACGAGGTTGTCGACTACGGTTACGGCGCCGTCTCCAAGGGCACGTCAAGTCCCTATCTTCCGTTTGGTGC : 1900
cyp51A_Env7  : AGAACCAGGCTACTAAGGAGCAGGAGAACGACGAGGTTGTCGACTACGGTTACGGCGCCGTCTCCAAGGGCACGTCAAGTCCCTATCTTCCGTTTGGTGC : 1900
cyp51A_Env8  : AGAACCAGGCTACTAAGGAGCAGGAGAACGACGAGGTTGTCGACTACGGTTACGGCGCCGTCTCCAAGGGCACGTCAAGTCCCTATCTTCCGTTTGGTGC : 1900
cyp51A_Env9  : AGAACCAGGCTACTAAGGAGCAGGAGAACGACGAGGTTGTCGACTACGGTTACGGCGCCGTCTCCAAGGGCACGTCAAGTCCCTATCTTCCGTTTGGTGC : 1900
cyp51A_Env10 : AGAACCAGGCTACTAAGGAGCAGGAGAACGACGAGGTTGTCGACTACGGTTACGGCGCCGTCTCCAAGGGCACGTCAAGTCCCTATCTTCCGTTTGGTGC : 1900

cyp51A_Env1  : TGGCCGACACCGCTGTATCGGCGAGAAATTCGCTTATGTCAACCTTGGTGTGATTCTGGCGACCATTGTGCGCCACCTGCGACTTTTCAACGTGGATGGA : 1966
cyp51A_Env2  : TGGCCGACACCGCTGTATCGGCGAGAAATTCGCTTATGTCAACCTTGGTGTGATTCTGGCGACCATTGTGCGCCACCTGCGACTTTTCAACGTGGATGGA : 1966
cyp51A_Env3  : TGGCCGACACCGCTGTATCGGCGAGAAATTCGCTTATGTCAACCTTGGTGTGATTCTGGCGACCATTGTGCGCCACCTGCGACTTTTCAACGTGGATGGA : 1966
cyp51A_Env4  : TGGCCGACACCGCTGTATCGGCGAGAAATTCGCTTATGTCAACCTTGGTGTGATTCTGGCGACCATTGTGCGCCACCTGCGACTTTTCAACGTGGATGGA : 1966
cyp51A_Env5  : TGGCCGACACCGCTGTATCGGCGAGAAATTCGCTTATGTCAACCTTGGTGTGATTCTGGCGACCATTGTGCGCCACCTGCGACTTTTCAACGTGGATGGA : 1966
cyp51A_Env6  : TGGCCGACACCGCTGTATCGGCGAGAAATTCGCTTATGTCAACCTTGGTGTGATTCTGGCGACCATTGTGCGCCACCTGCGACTTTTCAACGTGGATGGA : 2000
cyp51A_Env7  : TGGCCGACACCGCTGTATCGGCGAGAAATTCGCTTATGTCAACCTTGGTGTGATTCTGGCGACCATTGTGCGCCACCTGCGACTTTTCAACGTGGATGGA : 2000
cyp51A_Env8  : TGGCCGACACCGCTGTATCGGCGAGAAATTCGCTTATGTCAACCTTGGTGTGATTCTGGCGACCATTGTGCGCCACCTGCGACTTTTCAACGTGGATGGA : 2000
cyp51A_Env9  : TGGCCGACACCGCTGTATCGGCGAGAAATTCGCTTATGTCAACCTTGGTGTGATTCTGGCGACCATTGTGCGCCACCTGCGACTTTTCAACGTGGATGGA : 2000
cyp51A_Env10 : TGGCCGACACCGCTGTATCGGCGAGAAATTCGCTTATGTCAACCTTGGTGTGATTCTGGCGACCATTGTGCGCCACCTGCGACTTTTCAACGTGGATGGA : 2000
```

```
                                                                                                            S
cyp51A_Env1  : AAGAAAGGAGTCCCTGAAACTGACTATTCATCCCTCTTTTCGGGCCCCATGAAGCCAAGCATCATCGGCTGGGAGAAGCGGTCGAAAAACACATCCAAGT : 2066
cyp51A_Env2  : AAGAAAGGAGTCCCTGAAACTGACTATTCATCCCTCTTTTCGGGCCCCATGAAGCCAAGCATCATCGGCTGGGAGAAGCGGTCGAAAAACACATCCAAGT : 2066
cyp51A_Env3  : AAGAAAGGAGTCCCTGAAACTGACTATTCATCCCTCTTTTCGGGCCCCATGAAGCCAAGCATCATCGGCTGGGAGAAGCGGTCGAAAAACACATCCAAGT : 2066
cyp51A_Env4  : AAGAAAGGAGTCCCTGAAACTGACTATTCATCCCTCTTTTCGGGCCCCATGAAGCCAAGCATCATCGGCTGGGAGAAGCGGTCGAAAAACACATCCAAGT : 2066
cyp51A_Env5  : AAGAAAGGAGTCCCTGAAACTGACTATTCATCCCTCTTTTCGGGCCCCATGAAGCCAAGCATCATCGGCTGGGAGAAGCGGTCGAAAAACACATCCAAGT : 2066
cyp51A_Env6  : AAGAAAGGAGTCCCTGAAACTGACTATTCATCCCTCTTTTCGGGCCCCATGAAGCCAAGCATCATCGGCTGGGAGAAGCGGTCGAAAAACACATCCAAGT : 2100
cyp51A_Env7  : AAGAAAGGAGTCCCTGAAACTGACTATTCATCCCTCTTTTCGGGCCCCATGAAGCCAAGCATCATCGGCTGGGAGAAGCGGTCGAAAAACACATCCAAGT : 2100
cyp51A_Env8  : AAGAAAGGAGTCCCTGAAACTGACTATTCATCCCTCTTTTCGGGCCCCATGAAGCCAAGCATCATCGGCTGGGAGAAGCGGTCGAAAAACACATCCAAGT : 2100
cyp51A_Env9  : AAGAAAGGAGTCCCTGAAACTGACTATTCATCCCTCTTTTCGGGCCCCATGAAGCCAAGCATCATCGGCTGGGAGAAGCGGTCGAAAAACACATCCAAGT : 2100
cyp51A_Env10 : AAGAAAGGAGTCCCTGAAACTGACTATTCATCCCTCTTTTCGGGCCCCATGAAGCCAAGCATCATCGGCTGGGAGAAGCGGTCGAAAAACACATCCAAGT : 2100

                                           TGGTGTGATCGGAATAGGTGTA   rc of cyp51A_Rev
cyp51A_Env1  : GAGACTGTTGTAACCATCGAGGACTTCAAAGGATTTGGTGT : 2107
cyp51A_Env2  : GAGACTGTTGTAACCATCGAGGACTTCAAAGGATTTGGTGT : 2107
cyp51A_Env3  : GAGACTGTTGTAACCATCGAGGACTTCAAAGGATTTGGTGT : 2107
cyp51A_Env4  : GAGACTGTTGTAACCATCGAGGACTTCAAAGGATTTGGTGT : 2107
cyp51A_Env5  : GAGACTGTTGTAACCATCGAGGACTTCAAAGGATTTGGTGT : 2107
cyp51A_Env6  : GAGACTGTTGTAACCATCGAGGACTTCAAAGGATTTGGTGT : 2141
cyp51A_Env7  : GAGACTGTTGTAACCATCGAGGACTTCAAAGGATTTGGTGT : 2141
cyp51A_Env8  : GAGACTGTTGTAACCATCGAGGACTTCAAAGGATTTGGTGT : 2141
cyp51A_Env9  : GAGACTGTTGTAACCATCGAGGACTTCAAAGGATTTGGTGT : 2141
cyp51A_Env10 : GAGACTGTTGTAACCATCGAGGACTTCAAAGGATTTGGTGT : 2141
```

Figure S3. Alignment of the isolated *cyp51B* nucleotide sequences. Location of primers cyp51B_Fw and cyp51B_Rev is indicated. Introns are represented by black bars above the alignment and were assigned according to the known splicing pattern for *cyp51B*. Black background colour indicates positions where identical nucleotides are present.

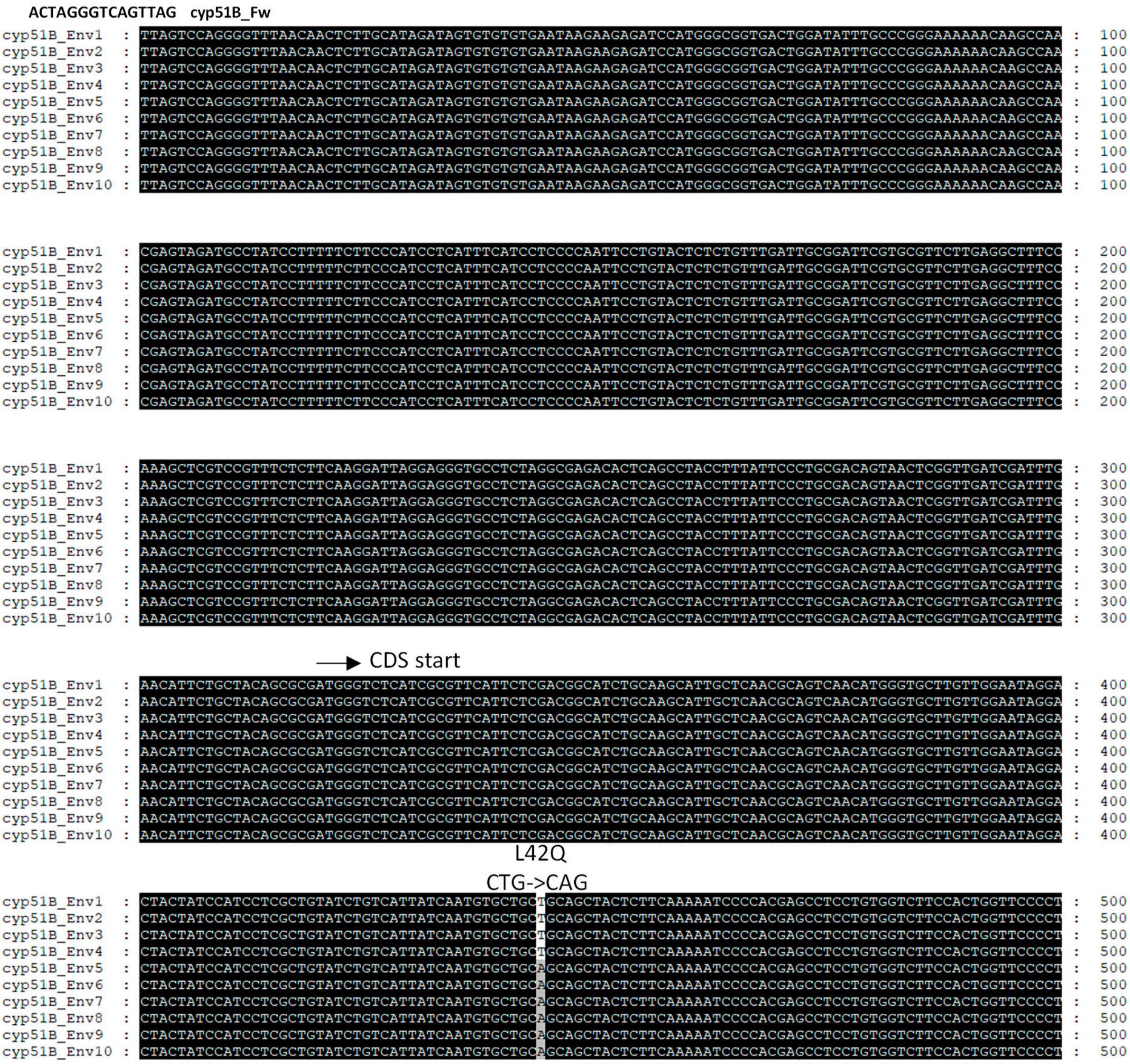

```
cyp51B_Env1  : TCATTGGTAGCACCATCAGTTATGGCATCGATCCGTACAAGTTCTTTTTCGACTGCCGCGCAAAGGTTTGCATTGTCGCTGCCAAATTTATGTGTCTAGC :  600
cyp51B_Env2  : TCATTGGTAGCACCATCAGTTATGGCATCGATCCGTACAAGTTCTTTTTCGACTGCCGCGCAAAGGTTTGCATTGTCGCTGCCAAATTTATGTGTCTAGC :  600
cyp51B_Env3  : TCATTGGTAGCACCATCAGTTATGGCATCGATCCGTACAAGTTCTTTTTCGACTGCCGCGCAAAGGTTTGCATTGTCGCTGCCAAATTTATGTGTCTAGC :  600
cyp51B_Env4  : TCATTGGTAGCACCATCAGTTATGGCATCGATCCGTACAAGTTCTTTTTCGACTGCCGCGCAAAGGTTTGCATTGTCGCTGCCAAATTTATGTGTCTAGC :  600
cyp51B_Env5  : TCATTGGTAGCACCATCAGTTATGGCATCGATCCGTACAAGTTCTTTTTCGACTGCCGCGCAAAGGTTTGCATTGTCGCTGCCAAATTTATGTGTCTAGC :  600
cyp51B_Env6  : TCATTGGTAGCACCATCAGTTATGGCATCGATCCGTACAAGTTCTTTTTCGACTGCCGCGCAAAGGTTTGCATTGTCGCTGCCAAATTTATGTGTCTAGC :  600
cyp51B_Env7  : TCATTGGTAGCACCATCAGTTATGGCATCGATCCGTACAAGTTCTTTTTCGACTGCCGCGCAAAGGTTTGCATTGTCGCTGCCAAATTTATGTGTCTAGC :  600
cyp51B_Env8  : TCATTGGTAGCACCATCAGTTATGGCATCGATCCGTACAAGTTCTTTTTCGACTGCCGCGCAAAGGTTTGCATTGTCGCTGCCAAATTTATGTGTCTAGC :  600
cyp51B_Env9  : TCATTGGTAGCACCATCAGTTATGGCATCGATCCGTACAAGTTCTTTTTCGACTGCCGCGCAAAGGTTTGCATTGTCGCTGCCAAATTTATGTGTCTAGC :  600
cyp51B_Env10 : TCATTGGTAGCACCATCAGTTATGGCATCGATCCGTACAAGTTCTTTTTCGACTGCCGCGCAAAGGTTTGCATTGTCGCTGCCAAATTTATGTGTCTAGC :  600

cyp51B_Env1  : TAACCAAATTTGCTTTTTGGCAGTATGGCGATATCTTCACATTCATTCTACTTGGCAAAAAGACAACGGTGTATCTTGGTACGAAGGGCAATGACTTCAT :  700
cyp51B_Env2  : TAACCAAATTTGCTTTTTGGCAGTATGGCGATATCTTCACATTCATTCTACTTGGCAAAAAGACAACGGTGTATCTTGGTACGAAGGGCAATGACTTCAT :  700
cyp51B_Env3  : TAACCAAATTTGCTTTTTGGCAGTATGGCGATATCTTCACATTCATTCTACTTGGCAAAAAGACAACGGTGTATCTTGGTACGAAGGGCAATGACTTCAT :  700
cyp51B_Env4  : TAACCAAATTTGCTTTTTGGCAGTATGGCGATATCTTCACATTCATTCTACTTGGCAAAAAGACAACGGTGTATCTTGGTACGAAGGGCAATGACTTCAT :  700
cyp51B_Env5  : TAACCAAATTTGCTTTTTGGCAGTATGGCGATATCTTCACATTCATTCTACTTGGCAAAAAGACAACGGTGTATCTTGGTACGAAGGGCAATGACTTCAT :  700
cyp51B_Env6  : TAACCAAATTTGCTTTTTGGCAGTATGGCGATATCTTCACATTCATTCTACTTGGCAAAAAGACAACGGTGTATCTTGGTACGAAGGGCAATGACTTCAT :  700
cyp51B_Env7  : TAACCAAATTTGCTTTTTGGCAGTATGGCGATATCTTCACATTCATTCTACTTGGCAAAAAGACAACGGTGTATCTTGGTACGAAGGGCAATGACTTCAT :  700
cyp51B_Env8  : TAACCAAATTTGCTTTTTGGCAGTATGGCGATATCTTCACATTCATTCTACTTGGCAAAAAGACAACGGTGTATCTTGGTACGAAGGGCAATGACTTCAT :  700
cyp51B_Env9  : TAACCAAATTTGCTTTTTGGCAGTATGGCGATATCTTCACATTCATTCTACTTGGCAAAAAGACAACGGTGTATCTTGGTACGAAGGGCAATGACTTCAT :  700
cyp51B_Env10 : TAACCAAATTTGCTTTTTGGCAGTATGGCGATATCTTCACATTCATTCTACTTGGCAAAAAGACAACGGTGTATCTTGGTACGAAGGGCAATGACTTCAT :  700

cyp51B_Env1  : CCTAAACGGCAAGCTGCGAGATGTTTGCGCTGAGGAGGTGTATTCTCCACTCACTACGCCTGTTTTTGGTCGACATGTTGTGTATGACTGCCCTAATGCT :  800
cyp51B_Env2  : CCTAAACGGCAAGCTGCGAGATGTTTGCGCTGAGGAGGTGTATTCTCCACTCACTACGCCTGTTTTTGGTCGACATGTTGTGTATGACTGCCCTAATGCT :  800
cyp51B_Env3  : CCTAAACGGCAAGCTGCGAGATGTTTGCGCTGAGGAGGTGTATTCTCCACTCACTACGCCTGTTTTTGGTCGACATGTTGTGTATGACTGCCCTAATGCT :  800
cyp51B_Env4  : CCTAAACGGCAAGCTGCGAGATGTTTGCGCTGAGGAGGTGTATTCTCCACTCACTACGCCTGTTTTTGGTCGACATGTTGTGTATGACTGCCCTAATGCT :  800
cyp51B_Env5  : CCTAAACGGCAAGCTGCGAGATGTTTGCGCTGAGGAGGTGTATTCTCCACTCACTACGCCTGTTTTTGGTCGACATGTTGTGTATGACTGCCCTAATGCT :  800
cyp51B_Env6  : CCTAAACGGCAAGCTGCGAGATGTTTGCGCTGAGGAGGTGTATTCTCCACTCACTACGCCTGTTTTTGGTCGACATGTTGTGTATGACTGCCCTAATGCT :  800
cyp51B_Env7  : CCTAAACGGCAAGCTGCGAGATGTTTGCGCTGAGGAGGTGTATTCTCCACTCACTACGCCTGTTTTTGGTCGACATGTTGTGTATGACTGCCCTAATGCT :  800
cyp51B_Env8  : CCTAAACGGCAAGCTGCGAGATGTTTGCGCTGAGGAGGTGTATTCTCCACTCACTACGCCTGTTTTTGGTCGACATGTTGTGTATGACTGCCCTAATGCT :  800
cyp51B_Env9  : CCTAAACGGCAAGCTGCGAGATGTTTGCGCTGAGGAGGTGTATTCTCCACTCACTACGCCTGTTTTTGGTCGACATGTTGTGTATGACTGCCCTAATGCT :  800
cyp51B_Env10 : CCTAAACGGCAAGCTGCGAGATGTTTGCGCTGAGGAGGTGTATTCTCCACTCACTACGCCTGTTTTTGGTCGACATGTTGTGTATGACTGCCCTAATGCT :  800

cyp51B_Env1  : AAGCTTATGGAGCAGAAGAAGGTAAGCGACTTTGGCAGAAACACTTTCGGCGACTAATAATCTTAGTTCGTCAAATACGGTCTCACCTCTGATGCGCTCC :  900
cyp51B_Env2  : AAGCTTATGGAGCAGAAGAAGGTAAGCGACTTTGGCAGAAACACTTTCGGCGACTAATAATCTTAGTTCGTCAAATACGGTCTCACCTCTGATGCGCTCC :  900
cyp51B_Env3  : AAGCTTATGGAGCAGAAGAAGGTAAGCGACTTTGGCAGAAACACTTTCGGCGACTAATAATCTTAGTTCGTCAAATACGGTCTCACCTCTGATGCGCTCC :  900
cyp51B_Env4  : AAGCTTATGGAGCAGAAGAAGGTAAGCGACTTTGGCAGAAACACTTTCGGCGACTAATAATCTTAGTTCGTCAAATACGGTCTCACCTCTGATGCGCTCC :  900
cyp51B_Env5  : AAGCTTATGGAGCAGAAGAAGGTAAGCGACTTTGGCAGAAACACTTTCGGCGACTAATAATCTTAGTTCGTCAAATACGGTCTCACCTCTGATGCGCTCC :  900
cyp51B_Env6  : AAGCTTATGGAGCAGAAGAAGGTAAGCGACTTTGGCAGAAACACTTTCGGCGACTAATAATCTTAGTTCGTCAAATACGGTCTCACCTCTGATGCGCTCC :  900
cyp51B_Env7  : AAGCTTATGGAGCAGAAGAAGGTAAGCGACTTTGGCAGAAACACTTTCGGCGACTAATAATCTTAGTTCGTCAAATACGGTCTCACCTCTGATGCGCTCC :  900
cyp51B_Env8  : AAGCTTATGGAGCAGAAGAAGGTAAGCGACTTTGGCAGAAACACTTTCGGCGACTAATAATCTTAGTTCGTCAAATACGGTCTCACCTCTGATGCGCTCC :  900
cyp51B_Env9  : AAGCTTATGGAGCAGAAGAAGGTAAGCGACTTTGGCAGAAACACTTTCGGCGACTAATAATCTTAGTTCGTCAAATACGGTCTCACCTCTGATGCGCTCC :  900
cyp51B_Env10 : AAGCTTATGGAGCAGAAGAAGGTAAGCGACTTTGGCAGAAACACTTTCGGCGACTAATAATCTTAGTTCGTCAAATACGGTCTCACCTCTGATGCGCTCC :  900

cyp51B_Env1  : GATCTTATGTTCCCTTAATTACGGATGAAGTAGAGAGCTTTGTCAAAAATTCTCCGGCGTTCCAGGGACACAAGGGCGTCTTCGATGTATGCAAAACAAT : 1000
cyp51B_Env2  : GATCTTATGTTCCCTTAATTACGGATGAAGTAGAGAGCTTTGTCAAAAATTCTCCGGCGTTCCAGGGACACAAGGGCGTCTTCGATGTATGCAAAACAAT : 1000
cyp51B_Env3  : GATCTTATGTTCCCTTAATTACGGATGAAGTAGAGAGCTTTGTCAAAAATTCTCCGGCGTTCCAGGGACACAAGGGCGTCTTCGATGTATGCAAAACAAT : 1000
cyp51B_Env4  : GATCTTATGTTCCCTTAATTACGGATGAAGTAGAGAGCTTTGTCAAAAATTCTCCGGCGTTCCAGGGACACAAGGGCGTCTTCGATGTATGCAAAACAAT : 1000
cyp51B_Env5  : GATCTTATGTTCCCTTAATTACGGATGAAGTAGAGAGCTTTGTCAAAAATTCTCCGGCGTTCCAGGGACACAAGGGCGTCTTCGATGTATGCAAAACAAT : 1000
cyp51B_Env6  : GATCTTATGTTCCCTTAATTACGGATGAAGTAGAGAGCTTTGTCAAAAATTCTCCGGCGTTCCAGGGACACAAGGGCGTCTTCGATGTATGCAAAACAAT : 1000
cyp51B_Env7  : GATCTTATGTTCCCTTAATTACGGATGAAGTAGAGAGCTTTGTCAAAAATTCTCCGGCGTTCCAGGGACACAAGGGCGTCTTCGATGTATGCAAAACAAT : 1000
cyp51B_Env8  : GATCTTATGTTCCCTTAATTACGGATGAAGTAGAGAGCTTTGTCAAAAATTCTCCGGCGTTCCAGGGACACAAGGGCGTCTTCGATGTATGCAAAACAAT : 1000
cyp51B_Env9  : GATCTTATGTTCCCTTAATTACGGATGAAGTAGAGAGCTTTGTCAAAAATTCTCCGGCGTTCCAGGGACACAAGGGCGTCTTCGATGTATGCAAAACAAT : 1000
cyp51B_Env10 : GATCTTATGTTCCCTTAATTACGGATGAAGTAGAGAGCTTTGTCAAAAATTCTCCGGCGTTCCAGGGACACAAGGGCGTCTTCGATGTATGCAAAACAAT : 1000
```

```
cyp51B_Env1  : CGCTGAAATCACCATTTACACTGCTTCGCGCTCGCTTCAAGGAAAAGAAGTTCGAAGCAAATTTGATTCCACATTCGCAGAGTTGTACCATAACCTTGAC : 1100
cyp51B_Env2  : CGCTGAAATCACCATTTACACTGCTTCGCGCTCGCTTCAAGGAAAAGAAGTTCGAAGCAAATTTGATTCCACATTCGCAGAGTTGTACCATAACCTTGAC : 1100
cyp51B_Env3  : CGCTGAAATCACCATTTACACTGCTTCGCGCTCGCTTCAAGGAAAAGAAGTTCGAAGCAAATTTGATTCCACATTCGCAGAGTTGTACCATAACCTTGAC : 1100
cyp51B_Env4  : CGCTGAAATCACCATTTACACTGCTTCGCGCTCGCTTCAAGGAAAAGAAGTTCGAAGCAAATTTGATTCCACATTCGCAGAGTTGTACCATAACCTTGAC : 1100
cyp51B_Env5  : CGCTGAAATCACCATTTACACTGCTTCGCGCTCGCTTCAAGGAAAAGAAGTTCGAAGCAAATTTGATTCCACATTCGCAGAGTTGTACCATAACCTTGAC : 1100
cyp51B_Env6  : CGCTGAAATCACCATTTACACTGCTTCGCGCTCGCTTCAAGGAAAAGAAGTTCGAAGCAAATTTGATTCCACATTCGCAGAGTTGTACCATAACCTTGAC : 1100
cyp51B_Env7  : CGCTGAAATCACCATTTACACTGCTTCGCGCTCGCTTCAAGGAAAAGAAGTTCGAAGCAAATTTGATTCCACATTCGCAGAGTTGTACCATAACCTTGAC : 1100
cyp51B_Env8  : CGCTGAAATCACCATTTACACTGCTTCGCGCTCGCTTCAAGGAAAAGAAGTTCGAAGCAAATTTGATTCCACATTCGCAGAGTTGTACCATAACCTTGAC : 1100
cyp51B_Env9  : CGCTGAAATCACCATTTACACTGCTTCGCGCTCGCTTCAAGGAAAAGAAGTTCGAAGCAAATTTGATTCCACATTCGCAGAGTTGTACCATAACCTTGAC : 1100
cyp51B_Env10 : CGCTGAAATCACCATTTACACTGCTTCGCGCTCGCTTCAAGGAAAAGAAGTTCGAAGCAAATTTGATTCCACATTCGCAGAGTTGTACCATAACCTTGAC : 1100

cyp51B_Env1  : ATGGGCTTCGCTCCTATCAACTTCATGTTACCCTGGGCCCCCCTACCCCACAACCGCAAAAGGGATGCTGCGCAGCGCAAATTGACCGAAACTTATATGG : 1200
cyp51B_Env2  : ATGGGCTTCGCTCCTATCAACTTCATGTTACCCTGGGCCCCCCTACCCCACAACCGCAAAAGGGATGCTGCGCAGCGCAAATTGACCGAAACTTATATGG : 1200
cyp51B_Env3  : ATGGGCTTCGCTCCTATCAACTTCATGTTACCCTGGGCCCCCCTACCCCACAACCGCAAAAGGGATGCTGCGCAGCGCAAATTGACCGAAACTTATATGG : 1200
cyp51B_Env4  : ATGGGCTTCGCTCCTATCAACTTCATGTTACCCTGGGCCCCCCTACCCCACAACCGCAAAAGGGATGCTGCGCAGCGCAAATTGACCGAAACTTATATGG : 1200
cyp51B_Env5  : ATGGGCTTCGCTCCTATCAACTTCATGTTACCCTGGGCCCCCCTACCCCACAACCGCAAAAGGGATGCTGCGCAGCGCAAATTGACCGAAACTTATATGG : 1200
cyp51B_Env6  : ATGGGCTTCGCTCCTATCAACTTCATGTTACCCTGGGCCCCCCTACCCCACAACCGCAAAAGGGATGCTGCGCAGCGCAAATTGACCGAAACTTATATGG : 1200
cyp51B_Env7  : ATGGGCTTCGCTCCTATCAACTTCATGTTACCCTGGGCCCCCCTACCCCACAACCGCAAAAGGGATGCTGCGCAGCGCAAATTGACCGAAACTTATATGG : 1200
cyp51B_Env8  : ATGGGCTTCGCTCCTATCAACTTCATGTTACCCTGGGCCCCCCTACCCCACAACCGCAAAAGGGATGCTGCGCAGCGCAAATTGACCGAAACTTATATGG : 1200
cyp51B_Env9  : ATGGGCTTCGCTCCTATCAACTTCATGTTACCCTGGGCCCCCCTACCCCACAACCGCAAAAGGGATGCTGCGCAGCGCAAATTGACCGAAACTTATATGG : 1200
cyp51B_Env10 : ATGGGCTTCGCTCCTATCAACTTCATGTTACCCTGGGCCCCCCTACCCCACAACCGCAAAAGGGATGCTGCGCAGCGCAAATTGACCGAAACTTATATGG : 1200

cyp51B_Env1  : AAATTATTAAGGCACGTCGTCAGGCTGGCAGTAAGAAGGATTCTGAAGACATGGTCTGGAACCTCATGTCCTGCGTTTACAAGAACGGCACGCCGGTTCC : 1300
cyp51B_Env2  : AAATTATTAAGGCACGTCGTCAGGCTGGCAGTAAGAAGGATTCTGAAGACATGGTCTGGAACCTCATGTCCTGCGTTTACAAGAACGGCACGCCGGTTCC : 1300
cyp51B_Env3  : AAATTATTAAGGCACGTCGTCAGGCTGGCAGTAAGAAGGATTCTGAAGACATGGTCTGGAACCTCATGTCCTGCGTTTACAAGAACGGCACGCCGGTTCC : 1300
cyp51B_Env4  : AAATTATTAAGGCACGTCGTCAGGCTGGCAGTAAGAAGGATTCTGAAGACATGGTCTGGAACCTCATGTCCTGCGTTTACAAGAACGGCACGCCGGTTCC : 1300
cyp51B_Env5  : AAATTATTAAGGCACGTCGTCAGGCTGGCAGTAAGAAGGATTCTGAAGACATGGTCTGGAACCTCATGTCCTGCGTTTACAAGAACGGCACGCCGGTTCC : 1300
cyp51B_Env6  : AAATTATTAAGGCACGTCGTCAGGCTGGCAGTAAGAAGGATTCTGAAGACATGGTCTGGAACCTCATGTCCTGCGTTTACAAGAACGGCACGCCGGTTCC : 1300
cyp51B_Env7  : AAATTATTAAGGCACGTCGTCAGGCTGGCAGTAAGAAGGATTCTGAAGACATGGTCTGGAACCTCATGTCCTGCGTTTACAAGAACGGCACGCCGGTTCC : 1300
cyp51B_Env8  : AAATTATTAAGGCACGTCGTCAGGCTGGCAGTAAGAAGGATTCTGAAGACATGGTCTGGAACCTCATGTCCTGCGTTTACAAGAACGGCACGCCGGTTCC : 1300
cyp51B_Env9  : AAATTATTAAGGCACGTCGTCAGGCTGGCAGTAAGAAGGATTCTGAAGACATGGTCTGGAACCTCATGTCCTGCGTTTACAAGAACGGCACGCCGGTTCC : 1300
cyp51B_Env10 : AAATTATTAAGGCACGTCGTCAGGCTGGCAGTAAGAAGGATTCTGAAGACATGGTCTGGAACCTCATGTCCTGCGTTTACAAGAACGGCACGCCGGTTCC : 1300

cyp51B_Env1  : CGATGAGGAAATCGCTCATATGATGATTGCCTTGTTGATGGCGGGTCAACATTCTTCCTCTTCTACTGCTTCCTGGATTGTTCTGCGCCTTGCGACACGG : 1400
cyp51B_Env2  : CGATGAGGAAATCGCTCATATGATGATTGCCTTGTTGATGGCGGGTCAACATTCTTCCTCTTCTACTGCTTCCTGGATTGTTCTGCGCCTTGCGACACGG : 1400
cyp51B_Env3  : CGATGAGGAAATCGCTCATATGATGATTGCCTTGTTGATGGCGGGTCAACATTCTTCCTCTTCTACTGCTTCCTGGATTGTTCTGCGCCTTGCGACACGG : 1400
cyp51B_Env4  : CGATGAGGAAATCGCTCATATGATGATTGCCTTGTTGATGGCGGGTCAACATTCTTCCTCTTCTACTGCTTCCTGGATTGTTCTGCGCCTTGCGACACGG : 1400
cyp51B_Env5  : CGATGAGGAAATCGCTCATATGATGATTGCCTTGTTGATGGCGGGTCAACATTCTTCCTCTTCTACTGCTTCCTGGATTGTTCTGCGCCTTGCGACACGG : 1400
cyp51B_Env6  : CGATGAGGAAATCGCTCATATGATGATTGCCTTGTTGATGGCGGGTCAACATTCTTCCTCTTCTACTGCTTCCTGGATTGTTCTGCGCCTTGCGACACGG : 1400
cyp51B_Env7  : CGATGAGGAAATCGCTCATATGATGATTGCCTTGTTGATGGCGGGTCAACATTCTTCCTCTTCTACTGCTTCCTGGATTGTTCTGCGCCTTGCGACACGG : 1400
cyp51B_Env8  : CGATGAGGAAATCGCTCATATGATGATTGCCTTGTTGATGGCGGGTCAACATTCTTCCTCTTCTACTGCTTCCTGGATTGTTCTGCGCCTTGCGACACGG : 1400
cyp51B_Env9  : CGATGAGGAAATCGCTCATATGATGATTGCCTTGTTGATGGCGGGTCAACATTCTTCCTCTTCTACTGCTTCCTGGATTGTTCTGCGCCTTGCGACACGG : 1400
cyp51B_Env10 : CGATGAGGAAATCGCTCATATGATGATTGCCTTGTTGATGGCGGGTCAACATTCTTCCTCTTCTACTGCTTCCTGGATTGTTCTGCGCCTTGCGACACGG : 1400

cyp51B_Env1  : CCTGATATAATGGAAGAGCTATATCAGGAACAAATTCGTGTTCTCGGATCTGACTTGCCTCCACTCACGTATGACAATCTGCAGAAGTTGGATTTGCACG : 1500
cyp51B_Env2  : CCTGATATAATGGAAGAGCTATATCAGGAACAAATTCGTGTTCTCGGATCTGACTTGCCTCCACTCACGTATGACAATCTGCAGAAGTTGGATTTGCACG : 1500
cyp51B_Env3  : CCTGATATAATGGAAGAGCTATATCAGGAACAAATTCGTGTTCTCGGATCTGACTTGCCTCCACTCACGTATGACAATCTGCAGAAGTTGGATTTGCACG : 1500
cyp51B_Env4  : CCTGATATAATGGAAGAGCTATATCAGGAACAAATTCGTGTTCTCGGATCTGACTTGCCTCCACTCACGTATGACAATCTGCAGAAGTTGGATTTGCACG : 1500
cyp51B_Env5  : CCTGATATAATGGAAGAGCTATATCAGGAACAAATTCGTGTTCTCGGATCTGACTTGCCTCCACTCACGTATGACAATCTGCAGAAGTTGGATTTGCACG : 1500
cyp51B_Env6  : CCTGATATAATGGAAGAGCTATATCAGGAACAAATTCGTGTTCTCGGATCTGACTTGCCTCCACTCACGTATGACAATCTGCAGAAGTTGGATTTGCACG : 1500
cyp51B_Env7  : CCTGATATAATGGAAGAGCTATATCAGGAACAAATTCGTGTTCTCGGATCTGACTTGCCTCCACTCACGTATGACAATCTGCAGAAGTTGGATTTGCACG : 1500
cyp51B_Env8  : CCTGATATAATGGAAGAGCTATATCAGGAACAAATTCGTGTTCTCGGATCTGACTTGCCTCCACTCACGTATGACAATCTGCAGAAGTTGGATTTGCACG : 1500
cyp51B_Env9  : CCTGATATAATGGAAGAGCTATATCAGGAACAAATTCGTGTTCTCGGATCTGACTTGCCTCCACTCACGTATGACAATCTGCAGAAGTTGGATTTGCACG : 1500
cyp51B_Env10 : CCTGATATAATGGAAGAGCTATATCAGGAACAAATTCGTGTTCTCGGATCTGACTTGCCTCCACTCACGTATGACAATCTGCAGAAGTTGGATTTGCACG : 1500
```
